# Smart5Grid Solutions for enhanced TSO grid observability and manageability in massive RES penetration environment

**DOI:** 10.12688/openreseurope.15090.1

**Published:** 2023-01-27

**Authors:** Daniel Shangov, Krassimir Vlachkov, Ralitsa Rumenova, Georgi Hristov, Atanas Velkov, Angelos Antonopoulos, Nicola Cadenelli, Nikolaos Tzanis, Dimitrios Brodimas, Michalis Rantopoulos, Ioannis Chochliouros, Vasiliki Vlahodimitropoulou

**Affiliations:** 1 Project Management, Elektroenergien Sistemen Operator (ESO EAD), Sofia, Bulgaria; 2 Entrea Energy, Sofia Tech Park, Bulgaria; 3 Bulgarian Telecommunications Company EAD, Sofia, Bulgaria; 4 Nearby Computing, Barcelona, Spain; 5 IPTO (ADMIE), Athens, Greece; 6 Hellenic Telecommunications Organization S.A. (OTE), Athens, Greece

**Keywords:** Smart Grid, 5G Open Experimentation Platform, Open Service Repository, Verification and Validation, NetApps

## Abstract

*Background *

This *article* elaborates on Horizon 2020 Smart5Grid Research and Innovation (R&I) *project* and reports the Smart5Grid Open Experimentation Platform (OEP), including its Open Service Repository (OSR), Verification and Validation (V&V), NetApp Controller (NAC), and Multi-access Edge Computing Orchestrator (MECO), as key results of this project. It then translates those results into energy vertical implications of the Smart5Grid for Transmission System Operators (TSOs), focusing on two particular Use Cases (UCs): UC3 Millisecond Level Precise Distribution Generation Control and UC4 Real-time Wide Area Monitoring pilot demonstrator of 5G virtual PDC capabilities for WAM of end-to-end electricity grids. More specifically, this work exhibits UC3 and UC4 NetApps developed as one of the key project deliverables.  All use cases generate openly accessible data, except where specific security restrictions are applicable.

*Methods *

The Smart5Grid development methodology is based on the concept of Network Applications (NetApps). Their main mission is to hide the complexity of a 5G telco network for energy application developers in a way that empowers them to develop a NetApp without having to deal with the underlying network. A Virtual Infrastructure Manager (VIM) such as OpenStack or Kubernetes hosts every unit that composes a NetApp. The VIM provides monitoring data to a Network Function Vurtualisation Manager and Orchestrator (NFV MANO) framework, which airs information to the NAC that employs analysis algorithms to propose the optimal Virtual Network Function (VNF) and NetApp placing. A Slice Manager (SM) reserves resources for all these capabilities.

*Results *

The Smart5Grid architecture represents the main result that this work delineates in the context of its enhanced 5G telecommunication provider (telco) capabilities for transmission and distribution grids to face and manage more efficiently high Renewable Energy Sources (RES) penetration in a decarbonisation context.

## Plain language summary

The purpose of this article is to delineate the Smart5Grid paradigm and its enhanced 5G telecommunication provider (telco) capabilities allowing transmission and distribution grids to face and manage more efficiently high Renewable Energy Sources (RES) penetration in a decarbonisation context. It touches upon 5G concepts of Information and Communication Technology (ICT) and how they could serve to optimize, enhance, and upgrade legacy Fiber Optic (FO) and Power Line Carrier (PLC) backbone communications commonly used in power grids to enable higher observability and manageability of Distributed Renewable Energy Resources (DRER). It also addresses leveraging Ultra Reliable Low-Latency Communication (URLLC) data exchange via the Smart5Grid platform for precise real-time Wide Area Monitoring (WAM) using measurements to counter preemptively inter-area frequency oscillations and power swings, boosting effective maintenance of TSO grid stability and reliability. Finally, key observations and conclusions are drawn on the Smart5Grid potential implications for the energy vertical from TSO perspective.

## Smart5Grid overview

The Smart5Grid (Demonstration of 5G solutions for SMART energy GRIDs of the future) project is intended to boost innovation for the highly critical and challenging energy vertical by providing an open 5G-enabled experimentation platform customized to support the smart grid vision.

**Figure 1.**
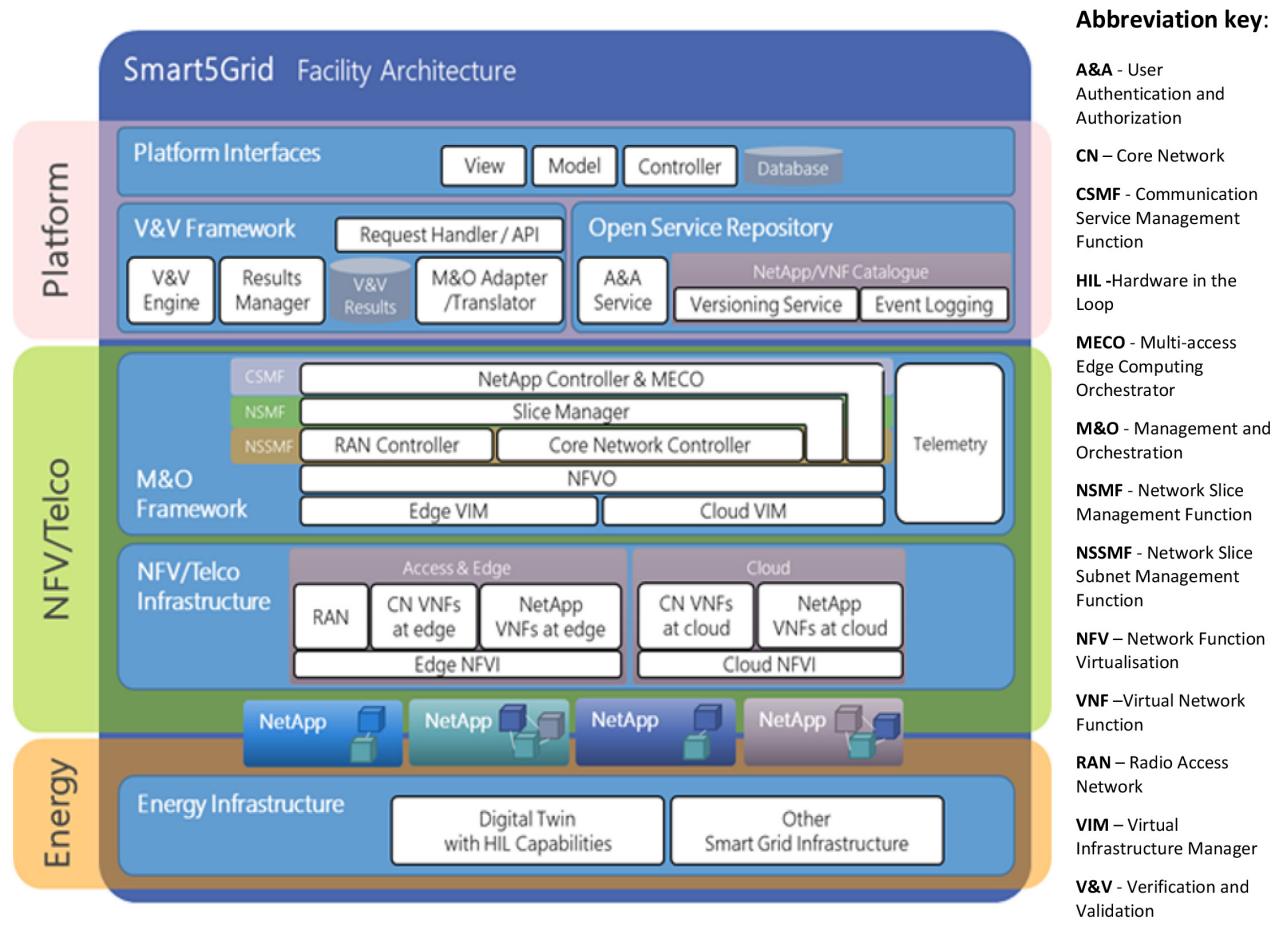
Overall design of the Smart5Grid Open-Service Experimentation Platform (OEP).

Specifically, as stated above, the main deliverable (result) of the Smart5Grid is the **Open 5G Experimentation Platform** ( Figure 1) enabling stakeholders in the energy vertical, ICT integrators, Network NetApp) developers, telecom industry actors, SMEs, and/or network service providers in general will be able to test, validate and share/expose their NetApps and create 5G open-source repositories for wide use and supporting standardisation.

### The Smart5Grid Open-Service Experimentation Platform

The **Open-Service Experimentation Platform (OEP)** is designed to enable applying and scaling-up Multi- Access Edge Computing (MEC) across the energy vertical, with particular focus on power grids. The main objective is to bring computation, storage, and network resources “closer” to the devices that make up power grids in order to solve inherent resource limitation issues and offload NetApps directly to MEC servers. This transpires into substantial reduction of E2E latency of devices accessing telco networks and, correspondingly, significant energy savings. MEC supports data security and integrity as well by enabling ubiquitous last-mile service access to the smart grid devices, while offering ultrafast and reliable deployment of network slices and value-added capabilities for the smart grid NetApps, such as bandwidth assurance, life cycles management of network services, and overall balancing of service loads.

As illustrated in Figure 1, the **Smart5Grid infrastructure consists of** three main interdependent and interfaced layers: (i) **Energy Infrastructure**; (ii) **Network Function Virtualization (NFV)/Telco**, and (iii) **the Open Experimentation Platform on top**. The platform itself is composed of **Open Service Repository (OSR), Verification and Validation (V&V) Framework, the NetApp Controller (NAC)**, and dedicated APIs (Application Programming Interfaces) and UIs (User Interfaces). The V&V Framework includes Verification and Validation (V&V) Engine, Results Manager, V&V Results container, and Management & Orchestration (M&O) Adapter and Translator. The OSR consists of an Authentication & Authorization (A&A) Service and NetApp/VNF Catalogue with Versioning Service and Event Logging components. This Catalogue serves to store ready (meaning verified and validated) NetApps that can be used by developers, for instance to assemble new NetApps. It is the OSR where NetApps are managed ( *i.e.*, upload, download, edit, delete, shared, *etc.*) following user identification (ID) and access routines. The V&V is where they undergo testing, verification, and validation prior to going live. As a key component of the platform, the NAC is architecturally located on system management level and will house the MEC offloading and Elastic VNF sizing and chaining functions. Its main responsibility is to save energy, optimize and accelerate data processing by proper parallelization and code partitioning of NetApps offloaded between the centralized platform and its MEC counterpart. Since latency minimisation is one of the key objectives of Smart5Grid, it is be met by one-dimensional search algorithm that follows an optimal offloading decision policy according to the application buffer queuing state, available processing powers of MEC servers, and characteristics of the Channel States through Channel State Information (CSI) between the MEC servers and the Phasor Measurement Units (PMUs) ^5^ and other metering devices.

The Energy infrastructure representing the platform’s bottom hierarchy layer in Figure 1 includes a digital twin of (part of) the power grid used for Hardware-in-the-loop (HIL) ^6^ experimentation and testing with RT data according to the concrete Use Case (UC) setup, *e.g.*, deriving from PMUs and virtual Phasor Data Concentrators (vPDC) ^7^. It also accommodates other smart grid hardware such as Intelligent Electronic Devices (IEDs), metering equipment, sensors, *etc.* This is where NetApps interface with the NFV/Telco layer consisting of Edge and Cloud Network Function Virtualization Infrastructure (NFVI), in turn composed of Radio Access Network (RAN) as well as edge and cloud Core Network (CN) and NetApp Virtual Network Functions (VNFs). The NFV/Telco layer itself is subordinated to the Management and Orchestration Framework (M&O) with its Communication Service Management Function (CSMF), Network Slice Management Function (NSMF), and Network Slice Subnet Management Function (NSSNF). These functions support, correspondingly, the NetApp Controller & Multi-access Edge Computing Orchestrator (MECO), the Slice Manager, and the RAN and CN Controllers interacting via Telemetry (data buses) with the Network Function Virtualization Orchestrator (NFVO) and the Virtual Machines (VIMs) of cloud and edge resources.

## OEP and Use Case Development Methods

The Smart5Grid OEP is developed by adaptable architectural design that builds an integrated infrastructure accommodating the entire spectrum of energy vertical’s communications and computational needs. The OEP **Verification and Validation** (V&V) framework is developed using API Handler based on Golang gin-gonic framework, its Pipeline engine is based on Argo Workflow ^8^, and its Results Manager uses Golang with MongoDB. The **Open Service Repository (OSR)** Authentication and Authorization Service is created using Python programming language, and the driver software implementation is based on Keycloak ^9^ open-source software for user management and single-sign-on (based on OpenID connect) for all OSR components and the OEP User Interface. The OSR Authentication and Authorization Service Python programming language is used for the driver software implementation.

Keycloak open-source software is used for user management and single-sign-on (based on OpenID connect) for all OSR components and the OEP User Interface. The OSR NetApp Catalogue Programming Code Language (and framework) is Python (Django), Database: PostgreSQL. The OSR Code Versioning Service is created using Python programming language for the driver software implementation Integration with GitLab ^10^ open-source software to store object descriptors and keep track of their different versions. The OSR Images Registries are also based on Python programming language for implemented driver software Integration with Harbor ^11^ open-source software to store container images and Helm charts. The OSR Event Logging Service is heavily based on Elasticsearch ^12^, Logstash ^13^, and Beat agents ^14^ open-source software. Elasticsearch is used as a distributed log database and search engine while Logstash is employed to modify logs to a unified format Beat agents in each logged component to gather and push logs to Logstash.

NetApps are the core concept of OEP. They handle the complexity of a 5G telco network so that energy application developers are only focused on developing a NetApp without having to deal with the underlying network. OpenStack or Kubernetes VIM is used to host every unit that forms a NetApp. The VIM feeds monitoring data to a NFV MANO framework. The latter broadcasts information to NAC that relies on analysis algorithms to propose the optimal VNF and NetApp placing. A Slice Manager performs optimal allocation of resources for all these capabilities. Using that reference design, Smart5Grid has created an integrated Development and Operations (DevOps) methodology manifested as a Verification and Validation (V&V) framework of NetApps and Network Services (NSs) as Virtual Network Functions (VNF) graphs enabling operators to monitor their behaviour.

Use Cases (UCs) 3 and 4, which this article refers to as demonstrators of Run Time Energy Production Monitoring and Predictive Maintenance (Enabler) for UC3 as well as virtual Phasor Data Concentrator (vPDC), Wide Area Monitoring (WAM), and Advisory (Enabler) for UC4, are developed by means of Grid Protection Alliance (GPA) ^15^ open source software and GSF open source library ^16^. Another two GPA open source projects used for both UCs development are openPDC ^17^ and openHistorian ^18^. Microsoft Visual Studio 2022 is the coding environment while C# и JavaScript are the programming languages engaged in the Use Cases 3&4 NetApp development process. Correspondingly, the services for both UCs are placed in Docker images that are used in Helm charts. These Helm charts are used to install the services in Kubernetes cluster, which runs Docker containers of the services in its cluster. Detailed video demonstrations of both use cases’ services are referred to in the results section of this paper. Figure 9 and Figure 10 also show the Graphical User Interface of Use Case 4 as key demonstrator from TSO perspective.

Regarding Use Case 3&4 trials, digital twin and Hardware-In-the-Loop (HIL) pre-piloting testing is as an advanced analytical and trial method that the project uses to simulate and investigate power system phenomena and components. Realistic and flexible experimentation and testing conditions for de-risking equipment are its salient benefits. The project’s methodology creates a common basis for testing and facilitating the analysis of RES integration at power distribution and transmission levels.

### Synopsis of the Smart5Grid Use Cases

**UC1: Automatic Power Distribution Grid Fault Detection (Italian demo)**: This represents a demo of public 5G RAN integrated monitoring for RT identification and insulation of faults in the backbone distribution network (self-healing) using an array of Intelligent Electronic Devices (IEDs) and a UC-specific NetApp to discriminate the source of a grid from a communication disturbance in a Distribution System Operator (DSO) (ENEL ^19^) self-healing automation system.

**UC2: Improved Safety of Maintenance Personnel by Automatically Delimited Working Areas at Distribution Level (Spanish demo)**: This UC demonstrates Improved safety of maintenance workers in HV power substation by virtual volumetric delimitation of working areas in a distribution substation of the Spanish DSO, e-distribucion ^20^.

**UC3: Millisecond level precise distribution generation control (Bulgarian demo)**: This UC will demonstrate precise monitoring of distributed generation at the millisecond level, which addresses the field of operation and maintenance of distributed power generation with a specific emphasis on renewable sources.

**UC4: Real-time wide area monitoring (Bulgarian-Greek demo)**: This UC will demonstrate a 5G solution for Wide Area Monitoring (WAM) of large interconnected power grids. For this purpose, 5G MEC vPDC capabilities will be used for experimentation WAM of end-to-end electricity grids through inter-TSO Regional Security Coordination (RCS).

UC3 and UC4 are discussed in detail below from TSO perspective within the main subject this article.

## Smart5Grid demos for enhanced observability and manageability of massive RES penetration from TSO perspective

Two of the four Smart5Grid use cases as described above, *i.e.*, UC3 and UC4, demonstrate how 5G Network 5G slicing capabilities such as eMMB (enhanced Mobile Broadband), URLLC (Ultra-Reliable and Low Latency Communications) and mMTC (massive Machine Type Communications) could provide TSOs with a high level of observability and manageability of Distributed Renewable Energy Recourses (DRERs). Since UC1 and UC3 address, correspondingly, self-healing and safety aspects at distribution level, this article only focuses on UC3 and UC4 with a particular view of technical (frequency response, voltage and load flow control) as well as market (electricity and balancing) implications of high-speed flexible 5G communications from TSO point of view.

## UC3: Millisecond level precise distributed generation monitoring

### Description and overall context

UC3 of the Smart5Grid project demonstrates precise monitoring of DRERs at millisecond level, which addresses the field of operation and maintenance of distributed power generation with a particular emphasis on renewable sources. More specifically, real-time (RT) monitoring of a wind farm, situated in southeast Bulgaria (Sliven area) and owned by Entra Energy ^21^, will be carried out using emerging capabilities of 5G telecom networks. RT monitoring is vital for the proper operation of wind farms for two key reasons: (i) the RES owner, being aware of the real-time status of its power asset, is able to predict and prevent in time potential future malfunctions that would cause significant financial losses; (ii) RES owners acting simultaneously as BRP (Balancing Responsible Party) and BSP (Balancing Service Provider) are responsible for potential imbalances and for providing real-time balancing services to the market. Precise RT high-granularity monitoring of electricity generation enables DRER owners to minimize their costs while meeting the standardized conditions for providing additional flexibility services (voltage, power, frequency and generation control, *etc.*) through flexible plant management.

UC3 demonstrates a viable DRER management solution for Wind Power Plants (WPPs) that could potentially be scaled up for other RES producers during the go-live phase following the end of the Smart5Grid project. The stringent requirements set by TSOs for the provision of RES services make it essential to use a highly reliable and secure communication between the physical asset (in this instance, a wind farm) and the TSO dispatch center. By using millisecond data exchange and native scalability of 5G technology, this facilitates generation forecast for balancing purposes, optimizes energy costs, and visualizes end users’ behavior in order to optimally manage their energy profile and provide flexibility services in the relevant electricity markets (intraday and balancing market) for multiple in nature (wind, solar, hydro) and geographically spread RES producers in the post project implementation phase.

The following example illustrates the benefits in terms of operational availability: wind farm (RES) owners or/and TSO are interested in being confident that the RES asset is currently in operation. Occasionally, maintenance, inspection, or unforeseen events may disrupt power plant operation. In such cases, should the plant manager omit notifying the TSO, this may lead to high balancing costs and, correspondingly, unbalanced penalties. RT monitoring can detect such errors in time, preventing escalation and allowing cost optimisation. As for flexibility service provision, the wind farm can deliver its operational data in real time to system operators (TSOs/DSOs), which enables flexible plant management with precise and reliable frequency and voltage control services by the TSO. It should be noted in this context that large WPPs have a communication line with the TSO’s SCADA, usually power line or FO. This is not the case for smaller, distributed WPPs such as the one we used in UC3. It is not currently incumbent on small WPPs to meet the same obligations as large WPPs. Nevertheless, this might and is likely to change in the future so that all distributed generation, irrespective of their size, are allowed to participate in the balancing market. In either instance, 5G offers flexibility and scalability at lower costs compared to FO, especially when it comes to remote locations where such communication infrastructure (FO) is not available in a reasonable proximity or is not feasible to deploy fast enough to accommodate RES penetration.

### Objectives of the UC3 demo

UC3 has three main business objectives: (i) render maintenance recommendations about the power plant (located in a rural area) based on RT measurements coming from multiple Internet-of-Things (IoT) sensors; (ii) support wind farm generation monitoring in hard real time, *i.e.*, millisecond resolution, and; (iii) provide wind farm owner with live monitor capabilities using a web dashboard and/or mobile app.

Those business objectives will be facilitated by four technical objectives: (i) collect RT measurements from an array of IoT sensors installed in the WPP; (ii) forecast wind farm generation to participate in day- ahead, intraday and balancing markets; (iii) collect RT generation to perform real-time control; and (iv) conduct data analysis to offer maintenance recommendations and RT analysis of wind farm performance.

### NetApps supporting the UC3 objectives

**Figure 2.**
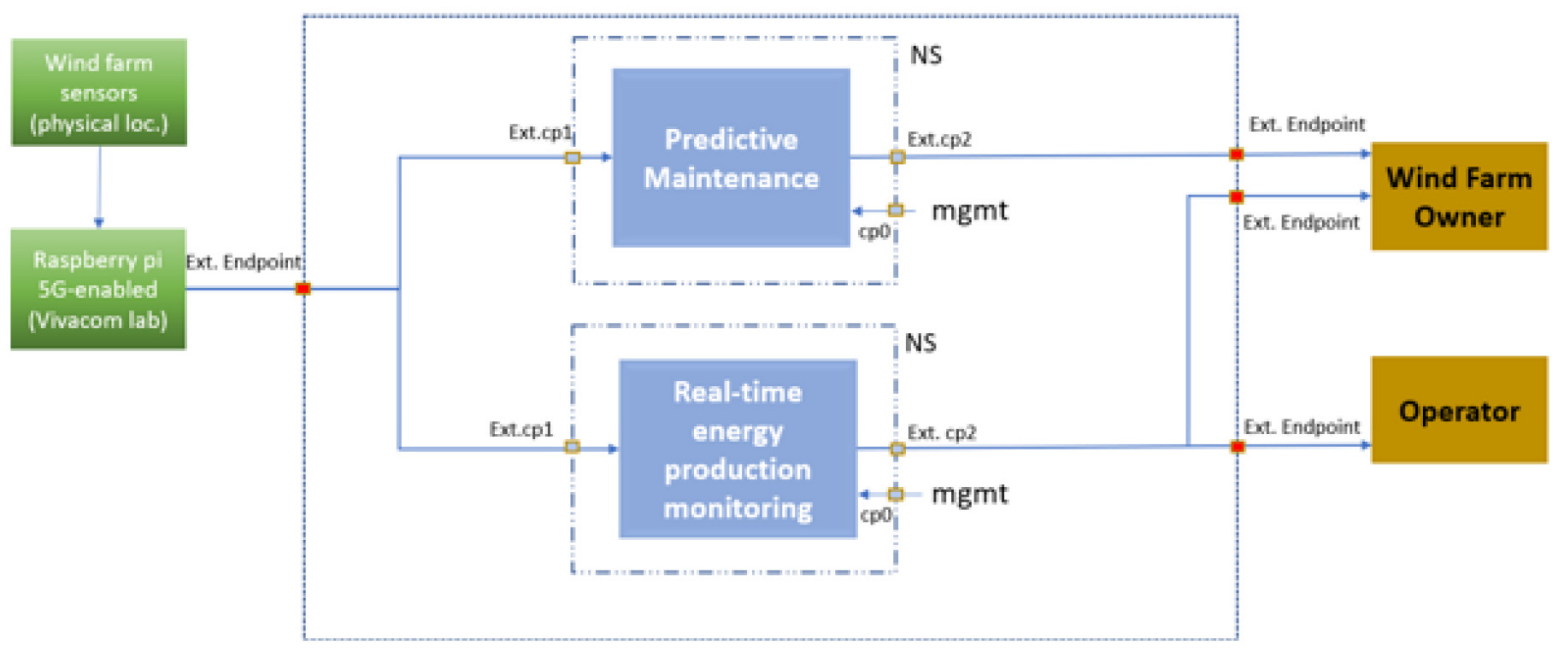
Use case 3 NetApp architecture.

There are two services (Virtual Network Functions, VNFs) intrinsic to UC3 and supported by one tailored NetApp whose architecture is illustrated in Figure 2: i) Predictive Maintenance Enabler (UC3 VNF1 based on mMTC) and ii) Real-Time Energy Production Monitoring (UC3 VNF2 based on URLLC). Each of these two components is implemented as a different VNF.

The preventive maintenance enabler (UC3 VNF1) has two external Connection Points (CPs), one being data input located in the telco ( VIVACOM) cloud to collect data from the Raspberry Pi signal emulator set up for UC3 to support 5G connectivity (5GHAT module), whilst the other one functions as data output transferring the result of internal processes to the farm owner (or to a 3 ^rd^ party maintenance service provider in the future). The real-time operation functionality (VNF2) also has two external CPs, one being data input located in the telco (VIVACOM) cloud to collect data from the Raspberry Pi signal emulator, whilst the other functions as data output transferring RT electricity generation signals to both the wind farm owner and the TSO. Both components feature control CPs to render access for setup or troubleshooting purposes.

The two specific VNFs of the UC3 NetApp operate as follows:

**UC3 VNF1 (Maintenance)** collects measurements from sensors installed on a 2MW wind turbine capturing the RT performance of key wind turbine components ( *e.g.*, generator, transformer, invertor, 20 kV switchgear). Then, it offers to the plant owner information on its generation parameters and serves as maintenance recommendations enabler by providing: (i) RT system alarms information to the asset owner, as well as; (ii) it serves as an enabler for building a NetAapp add-ons that will allow the 3 ^rd^ party providers ( *e.g.*, OEM-Original Equipment Manufacturers or Maintenance service providers) to leverage on this RT data and develop predictive maintenance algorithms and functionalities as part of the experimentation and post project market development phase.

**UC3 VNF2 (Monitoring)** provides RT data monitoring (in milliseconds from instrument transformer output) of wind farm ^22^ production to the power system owner and operator ( *i.e.*, TSO, in this specific instance the Bulgarian ESO EAD). By knowing RT production on a millisecond basis, TSOs are able to minimize total system costs while wind farm owners can benefit financially by providing innovative services to system operators (TSO, DSO or both depending on whose grid the wind farm is connected to), such as voltage and frequency control. In addition, it also enables transfer of RT operational data to DSO/TSO, informing them about the wind plant availability in real-time. In essence, both plant owners and TSO/DSOs monitor RES asset’s real-time generation in millisecond resolution. By using NetApp 1 capabilities, the wind farm owner can increase the efficiency and accuracy of generation control, forecasting and scheduling also using other information, such as weather data. On the other hand, the DSO/TSO can improve power system stability by monitoring RES output in hard real time.

**Figure 3.**
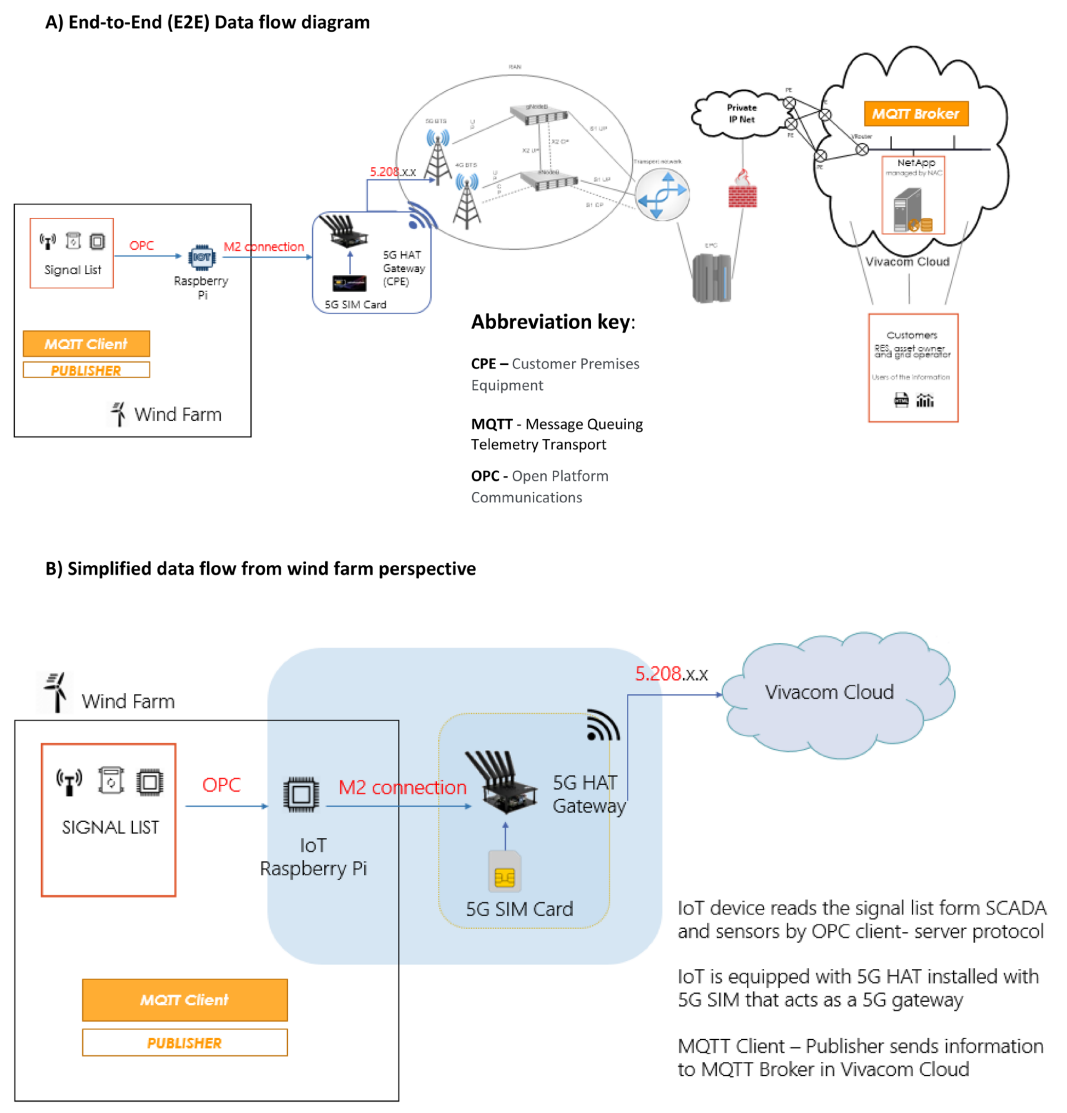
UC3 E2E Data Flow diagram with MQTT protocol for data transfer between wind turbine and NetApp.

Figure 3 illustrates the data flow between the wind farm’s IoT monitoring sensors and the UC3 NetApp functionalities using cloud-based Message Queuing Telemetry Transport (MQTT) Broker/Client servers on top of private Access Point Name (APN)/IP Network 5G architecture. Thousands of sensors (IoT devices) that measure electrical (voltage, current, frequency, active and reactive power), mechanical (turbine speed and vibration), and weather parameters (ambient temperature, humidity, and wind speed) are installed on turbine and feed RT measurement data to wind turbine owner’s local Supervisory Control and Data Acquisition (SCADA) . A 5G HAT equipped Raspberry Pi4 (Raspberry Pi 4 Computer Model B; BCM2711 SoC; 4GB DDR4 RAM; USB 3.0; PoE Enabled) reads those RT measurements (signal list) from the sensors and SCADA using OPC Client-Server protocol and broadcasts them through the 5G HAT gateway with 5G SIM to the VIVACOM Cloud. In this data flow configuration, MQTT Client – Publisher transfers signal list data to MQTT Broker in the VIVACOM Cloud from where the UC3 NetApps serve the Customers (RES asset owner andTSO/DSO) by delivering the millisecond monitoring functionalities of UC3. Demanding requirements for availability and reliability of communication services (as specified below for the UC3 demo) are met through adequate resilience and redundancy measures. This approach enables further and easy scalability across different types of RES producers (wind, hydro and solar) thus allowing the input from different type of sensors or plant SCADA systems to get connected and emulated using 5G IoT infrastructure in a reliable and secure manner.

RT measurements are communicated using the 5G infrastructure illustrated in Figure 3 to ensure reliable and secure operation of the wind farm and strengthen its Balancing Responsible Party (BRP) and Balancing Service Provider (BSP) capabilities. In a general perspective and taking into consideration the scalability potential, such solution generates benefits to TSOs in terms of higher visibility, predictability and controllability of renewable assets resulting in improved system balancing and security via ancillary services provided by RES owners.

**Main actors and their roles in the UC3 demo**: TSO in charge of system balancing and procurement of ancillary services; WPP owner; BSP tasked to deliver balancing services; BRP responsible for preventing imbalances in real-time market segment; Dispatching Center operating the TSO grid; IoT devises that measure RT operation of different WPP; UI display visualizing data according to user requirements; Telecommunication Provider that owns, operates and maintains the telco network (CN and RAN) and its main responsibility is to ensure stable and reliable 5G coverage meeting the 5G network specifications under the Service Level Agreement (SLA); Infrastructure Owner providing the server that will host the Smart5Grid platform as well as the NetApps running at the edge.

**Demo scenarios (operational algorithm)** for the energy vertical service delivered by the **UC3 NetApp**:

- No abnormalities in wind turbine operation detected, no alerts sent to WPP operator.
- Identified potential deviation of WPP operation in proximate future, maintenance projection sent to WPP owner (in case of predictive maintenance add-on is developed in the post project market implementation phase).
- RR WPP availability disruption (outage) detected, warning sent to both TSO and WPP owner.
- RT wind generation does not create system stability problem for TSO, no curtailment needed.
- RT wind generation causes system stability problem, TSO effects curtailment.

**5G network requirements for UC3 ^23^:** Ultra Reliable Low Latency Communication (URLLC) service availability and reliability of 99.999%, massive Machine Type Communication (mMTC) service availability and reliability of 99%, URLLC E2E latency of 20-200 msec, mMTC E2E latency not critical.

**Key Performance Indicators (KPIs) for the UC3 demo**: E2E Latency (minutes between user service and endpoints); reliability; average service creation time cycle reduced to 90 minutes.

With the 5G network data flow arrangement as shown in Figure 3, the actually measured E2E latency performance for UC3 is less than 10 msec, which compares to that of fiber optic or wire-bound network while offering additional flexibility in terms of network slicing in the form of URLLC and mMTC.

### Benefits of 5G deployment in the energy vertical as per UC3 scenario

**Business**: Increased observability of wind farm operations in terms of energy generation and life cycle of multi-parameter wind turbines enables RES owners to better manage their assets and offer innovative flexibility services.

**Economical**: Anticipatory preventive maintenance of RES assets means enhanced security of supply and optimized maintenance costs. By knowing RT generation with millisecond resolution, TSO is able to minimize overall system costs while RES asset owners enjoy financial benefits by providing the innovative services such as voltage control. In addition, optimized BRP management portfolio results in lower deviations from the set generation schedules and less need for, and costs of, balancing services.

**Social**: Secure and uninterrupted power supply to end consumers.

**Environmental**: High visibility of RES production improves power system control and reduces RES curtailment for balancing purposes. A higher share of RES in the energy mix transpires into clean energy production and lower carbon emission levels.

**Technological:** 5G is a relatively new technology and the research, testing and validation of IoT devices to operate over 5G in real-life use will pave the way for further deployment of IoT devices over 5G in the energy vertical as well as in other industrial domains.

## UC4: Real-time Wide Area Monitoring

### Description and overall context

UC4 is meant for real-time monitoring of a wide geographic area of synchronously interconnected power systems. It leverages 5G telco infrastructure to monitor electricity flows on existent 400 kV interconnector between Blagoevgrad (Bulgaria) and Thessaloniki (Greece) substations, focusing on system security coordination by the Regional Security Coordinator (RSC) in Thessaloniki, Greece. Both TSOs participating in this UC, *i.e.*, ESO EAD ^24^ and IPTO ^25^, are owners of the SEE RSC. In the Smart5Grid context, RSC is the primary actor although not directly involved as a partner in the project. RSC is considered as the WAM owner. RT power flow monitoring is indispensable for interconnected power system performance optimisation. In this context, UC4 is to improve the RSC live monitoring capabilities. This process uses PMUs located in Southern Bulgaria and Northern Greece to provide inputs (voltage, current and phase angle measurements) to an edge cloud-based (MEC server in telco data center) vPDC. In addition to collecting PMU measurements, the vPDC's role is to ensure that this data is synchronized and validated before being transferred to the RSC. Such time aligned PMU measurements provide high data granularity (sampling rate of 50 to 60 times per second, including positive, negative, and zero sequences of voltage and current). The use of 5G in UC4 provides URLLC connectivity between the PMUs and the vPDC, conforming strict network requirements for this UC, given its criticality for early detection of abnormalities such as power swings or frequency deviations and, consequently, enabling timely countermeasures to avoid disturbances and contingencies. The PMUs, vPDC and RSC arrangement forms the WAM system of the UC4 demo.

As DRERs expand at a high pace across Europe, the interconnected synchronous power systems become more complex and difficult to operate. With growing DRER penetration, inverter-connected devices gain on dominance, which results in lacking physical inertia. This, in turn, causes significant fluctuations in the Rate of Change of Frequency (RoCoF), leading to fundamental changes in system dynamics but also affecting electricity and balancing markets. It is therefore essential to have a WAM system capable of detecting and suppressing dynamic phenomena that create dangerous conditions for the stability of the entire synchronous European power system. Local disturbances can cause system-wide instability. WAM mainly benefits from the high PMU accuracy and the low latency of 5G mobile networks. The European synchronous power system has multiple control areas where each TSO is responsible for its own system control. For proper coordination between adjacent control areas, RSCs owned by neighbor TSOs are established. One of the five critical objectives of an RSC is coordinated security analysis across multiple timeframes (day ahead, intraday, and real time). For RT monitoring of their area (including areas controlled by multiple TSOs), RSCs provide advisory services to TSOs to facilitate disturbance-free system operation. In addition, RSCs offer ex-post information (following major network disturbances or frequency deviations) to the relevant TSOs to develop and improve guidance for this type of problem situation. In the context of this UC, the RSC RT monitoring function is demonstrated with PMU measurements from Southern Bulgaria and Northern Greece monitoring the interconnection area. The involved TSOs ( *i.e.*, ESO of Bulgaria and IPTO of Greece) then use the information about their connected assets and the recommendations from the RSC to better control and suppress events that could jeopardize system stability.

### Objectives of the UC4 demo

The UC4 is set to use a URLLC 5G pilot solution to monitor the status of PMUs and visualize their measurements in a way that enables the RSC to offer efficient advisory services to the Bulgarian and Greek TSOs, regarding power system management. Such measurements include voltage, current, phasors, and RoCoF on both sides of the monitored area. The combination of different features and the comparison of symmetrical features could also reveal hidden but useful results and conclusions. This will facilitate the business objective of having a regional power system that operates in secure conditions and is resilient to unusual dynamic contingencies that threaten the overall system balance.

### NetApp Supporting the UC4 Objectives

**Figure 4.**
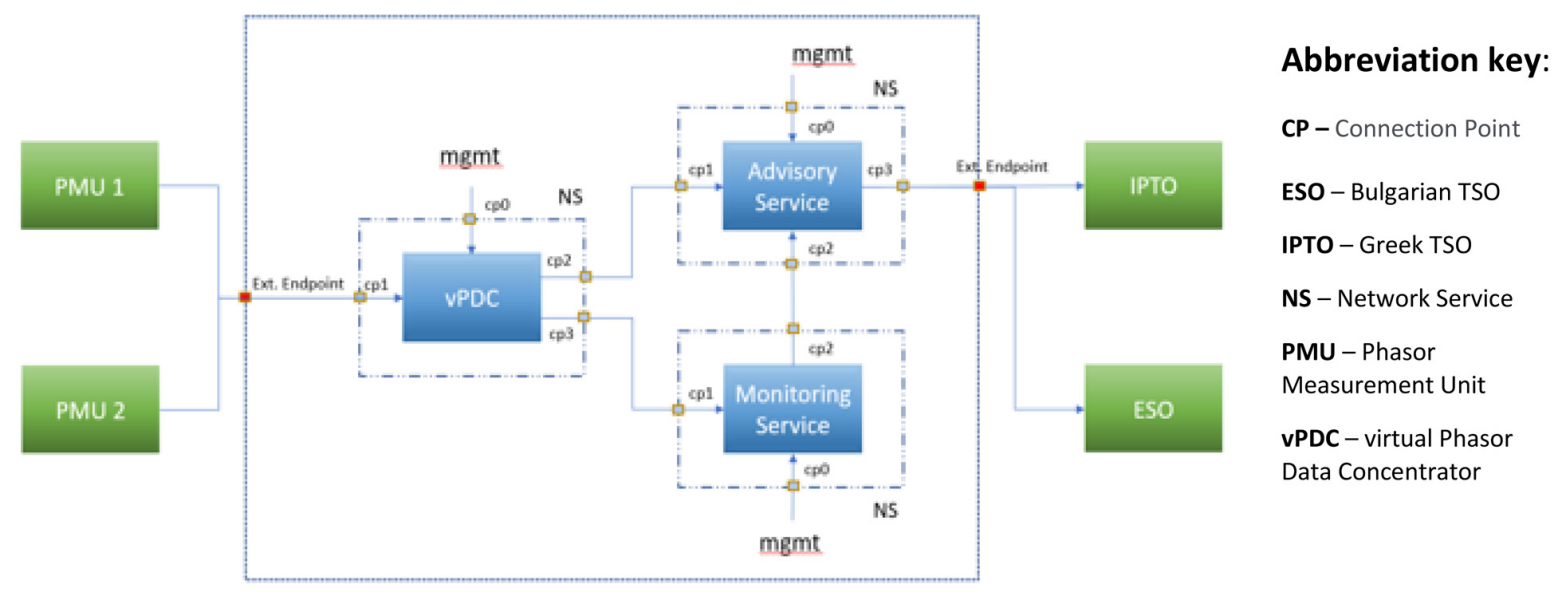
Use case 4 NetApp architecture.

The UC4 NetApp consists of three components (VNFs) whose architecture is illustrated in Figure 4, that is: vPDC, Monitoring Service, and Advisory Service. Each of these components is implemented as a different VNF that is linked and interacts with the other VNFs. Details on their design and creation methodology are provided in the OEP and Use Case Development section of this paper.

First, the vPDC VNF has two CPs for input from PMUs ^26^. Then, after data processing, the output goes to the next components *via* internal CPs. Next, the Monitoring VNF features an internal CP collecting the input from the vPDC, plus one CP to communicate with the Advisory VNF. Finally, the Advisory VNF has two internal CPs for input from the other two VNFs and one external CP to transfer advisory messages to both TSOs (ESO and IPTO).

All three NetApp components have control CPs to render access for setup and troubleshooting purposes. The Monitoring VNF’s control CP will also be used for the RSC (NetApp owner and administrator) to access visualized images to be displayed as part of the UC4 demo.

The UC4 NetApp VNFs operate as follows:

**UC4 VNF: vPDC** (URLLC) manages the vPDC service that collects comparable measurement data from the PMUs located in the wide area of Greece and Bulgaria. In addition to the effort of collecting data under the C37.118 protocol for comparison purposes, vPDC is specifically tasked to synchronize and validate measurements coming from different PMUs on both sides of the BG-GR (Bulgarian-Greek) border to deliver a real-time synchronized data stream. A considerable decrease of latency is achieved by virtualization of the PDC, which, *per se*, is closer to the PMUs and limits integration costs.

**UC4 VNF: WAM Service** (URLLC) builds on the vPDC to provide several PMU status indicators and visualization functions. These functions may include, *inter alia*: a map showing current location of the PMU; firmware name, address, model, serial number, and version; rated system frequency (Hz) and actual measured value (frames per second); current and voltage phasor graph with hard real time updates; voltage magnitude and angular difference monitor based on historical data for both monitored objects.

**UC4 VNF: Advisory Service** (URLLC) delivers RT consultancy system operation correction measures to the TSOs as well as for ex-post analysis in case of fault and contingency events.

**Figure 5.**
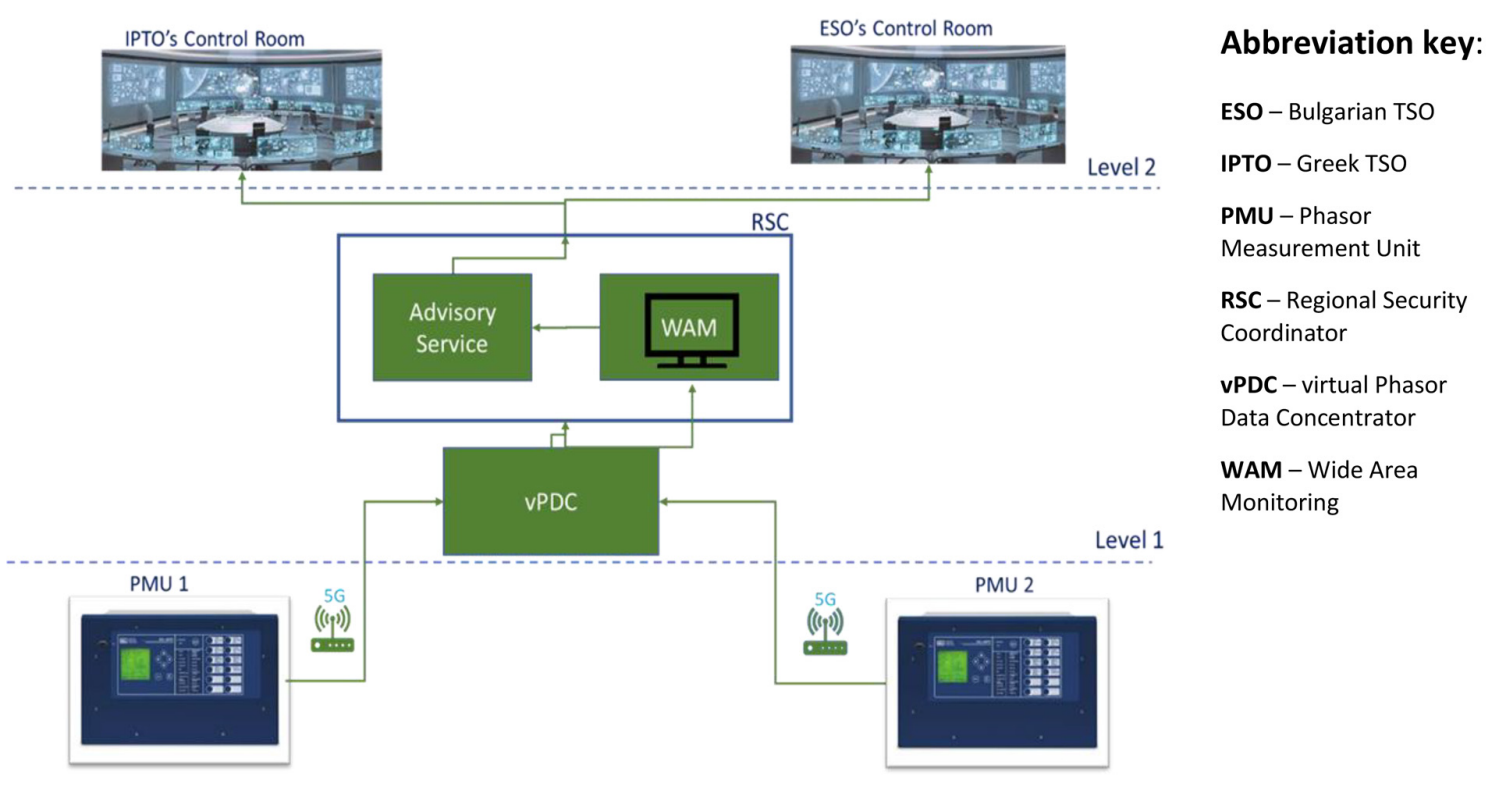
Use case 4 high-level diagram.

The high-level diagram for UC4 shown in Figure 5 illustrates the hierarchy interrelations between the energy infrastructure at Level 1 (PMUs installed in the wide area of Greece and Bulgaria), the edge cloud- based NetApp (MEC servers in telco data center), and the control rooms of both two TSOs at Level 2 where the three services will be provided.

### UC4 Scenarios and Sequence Diagrams

The UC4 sequence diagrams in Figure 6(a) and Figure 6(b) illustrate, correspondingly, the service instantiation and network connections between individual objects, on the one hand, and their operations once the service instantiation is completed, on the other hand.

**Figure 6a.**
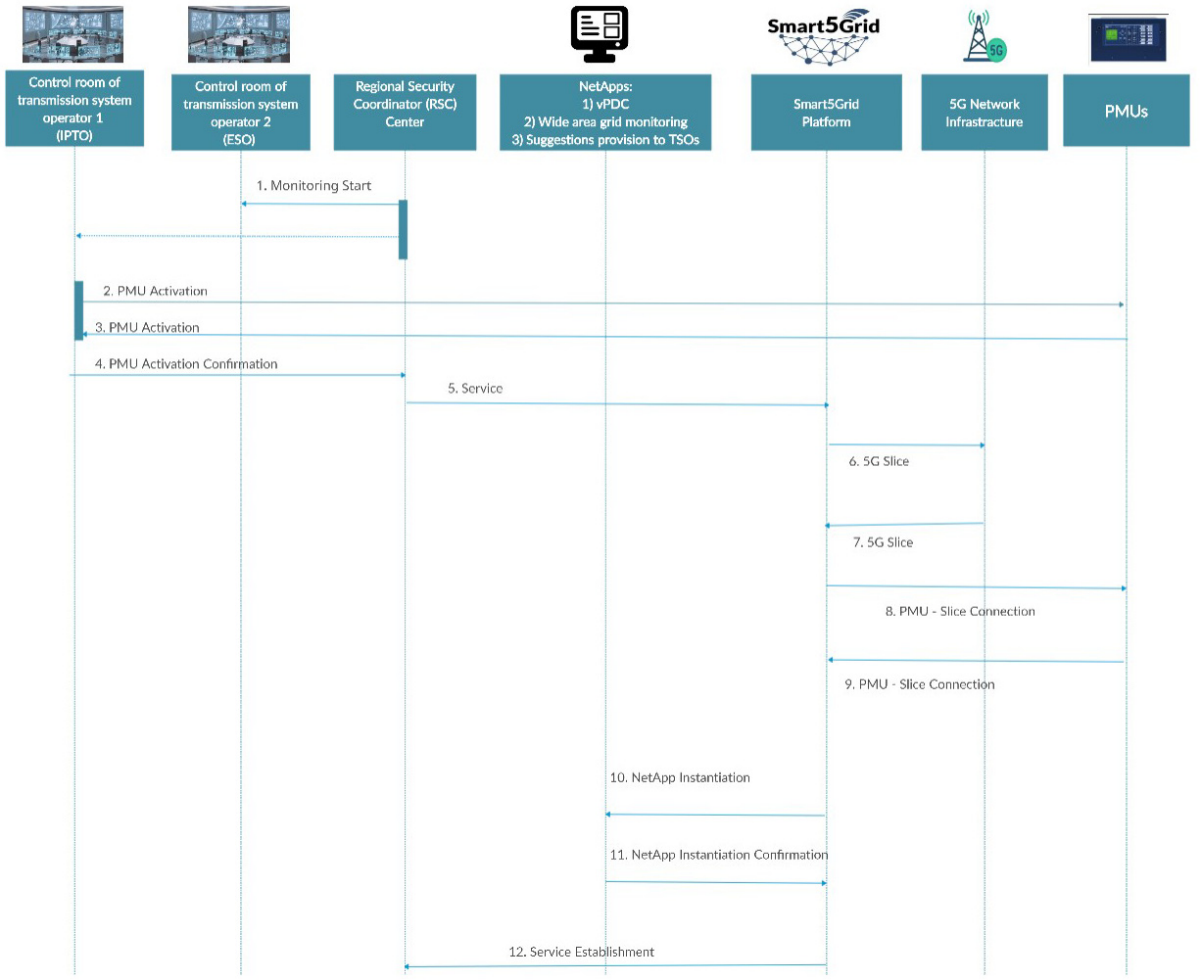
Use case (UC) 4 sequence diagram of Day-0 (instantiation).

**Figure 6b.**
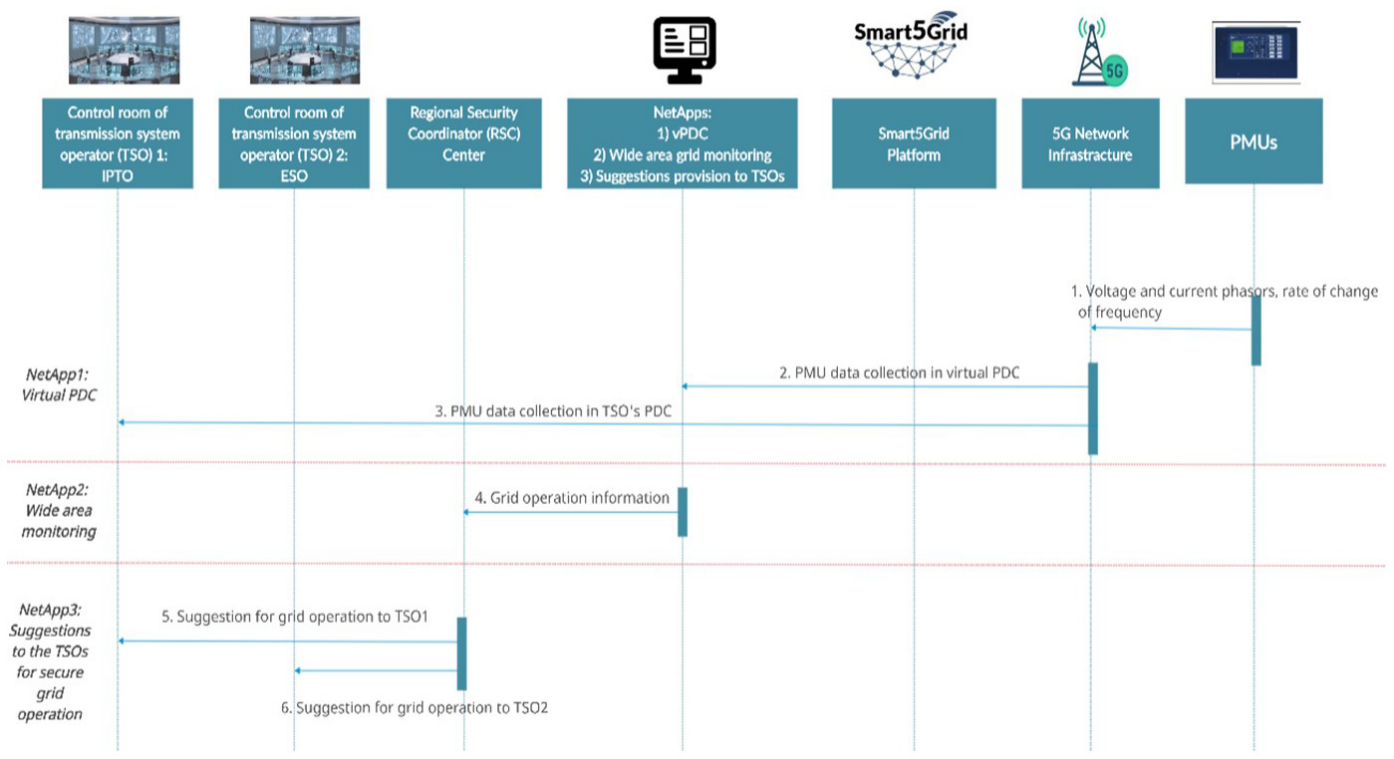
UC4 sequence diagram of an operational day.

The Day-0 sequence diagram in Figure 6(a) represents the UC4 service initiation (activation) consisting of the following steps:

1. RSC informs the transmission service operators that it will start the service.

2. IPTO requests PMU activation.

3. PMUs sent an activation confirmation signal to IPTO.

4. IPTO confirms PMU before RSC.

5. RSC sends service activation signal to the Smart5Grid platform.

6. The platform requests telco operator to provide 5G Network Slice.

7. Network Slice is provided.

8. The Smart5Grid platform informs PMUs about the Network Slice they should connect to.

9. PMUs confirm successful connection.

10. The Smart5Grid platform requests installation of NetApps.

11. Once installed, NetApps confirm activation.

12. The Smart5Grid platform informs that service have been initiated.

The sequence diagram of an operational day shown above has the following conceptual flow:

1. PMUs perform the necessary measurements and forward them to the 5G infrastructure.
2. Data packages transferred *via* the 5G infrastructure arrive at vPDC.
3. Data packages also arrive to the first TSO for its own use.
4. Synchronized data from vPDC arrive at RSC for visualization.
5. Advisory suggestions are delivered to the TSO depending on the type of abnormality found.
6. Same as step 5 for the second TSO.

All six steps are made in hard real time to enable the TSOs to counteract to rapidly evolving dynamic events in the synchronously interconnected power system.

**Main actors and their roles in the UC4 demo**: RSC is the primary owning the WAM and servicing both TSOs (ESO and IPTO): TSO owns the transmission grid assets and responsible for ensuring grid stability and Security of Supply (SoS); Telecommunication Provider Telecommunication Provider owns, operates and maintains the telco network (CN and RAN) and its main responsibility is to ensure stable and reliable 5G coverage meeting the SLA 5G network specifications; Infrastructure Owner providing the server that will host the Smart5Grid platform as well as the NetApps running at the edge; PMU measuring grid parameters ( *e.g.*, current and voltage magnitude and phase angle) with high-precision time synchronization.

**Demo scenarios** for the energy vertical service delivered by the **UC4 NetApp**:

- No system operation abnormalities found, no advisory suggestion offered.
- RoCoF deviation found, advisory suggestion sent to both TSOs.
- Voltage/current *phasor* abnormalities found, advisory suggestion sent to the affected TSO.

**5G network requirements for UC4:** URLLC service availability and reliability of 99.999%, URLLC E2E latency of 20-200 msec; vPDC absolute waiting time of 40 msec; Bandwith 699-1500 kbps/node ^27^ Device Density of 1 Dev/km; High Security

**Key Performance Indicators (KPIs) for the UC4 demo**: E2E Latency (min. between user service endpoints), Reliability, Bandwidth, Network Slicing (URLLC), and vPDC Absolute Waiting Time.

The actually measured E2E latency performance for UC4 is less than 10 msec, which compares to that of fiber optic or wire-bound network while offering additional flexibility in terms of network slicing in the form of URLLC.

**Figure 7.**
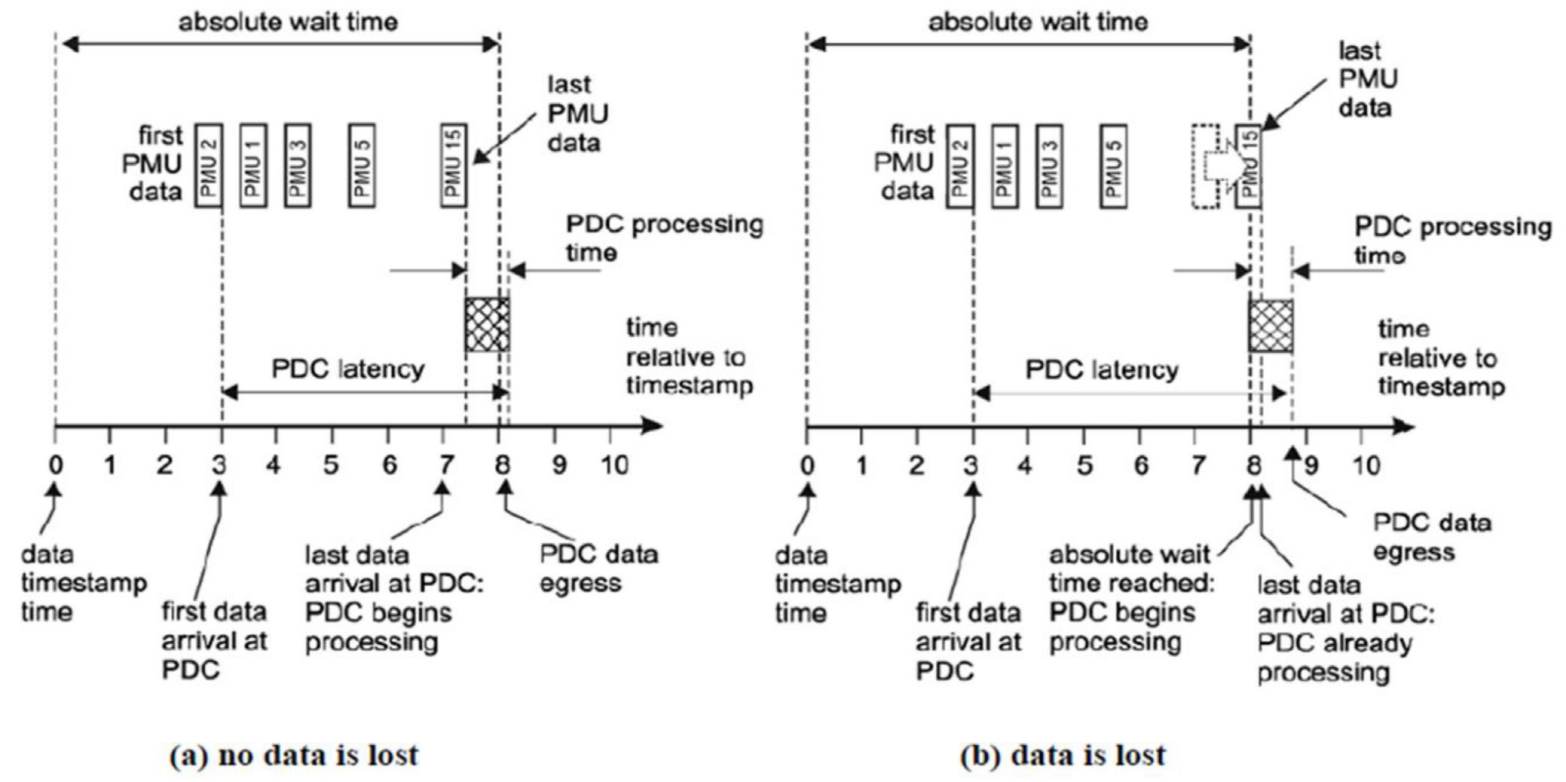
Example of PDC latency for data aggregation with alignment to absolute wait time (Source: Smart5Grid).

An indicative example of PDC latency for data aggregation with time alignment to absolute time is given in Figure 7. It illustrates that measurement packs arriving from the PMUs to the vPDC that will be processed (aggregation, synchronization and validation) without data loss as long as their arrival has been within the vPDC absolute wait time, and the latency limit is not exceeded.

**Figure 8.**
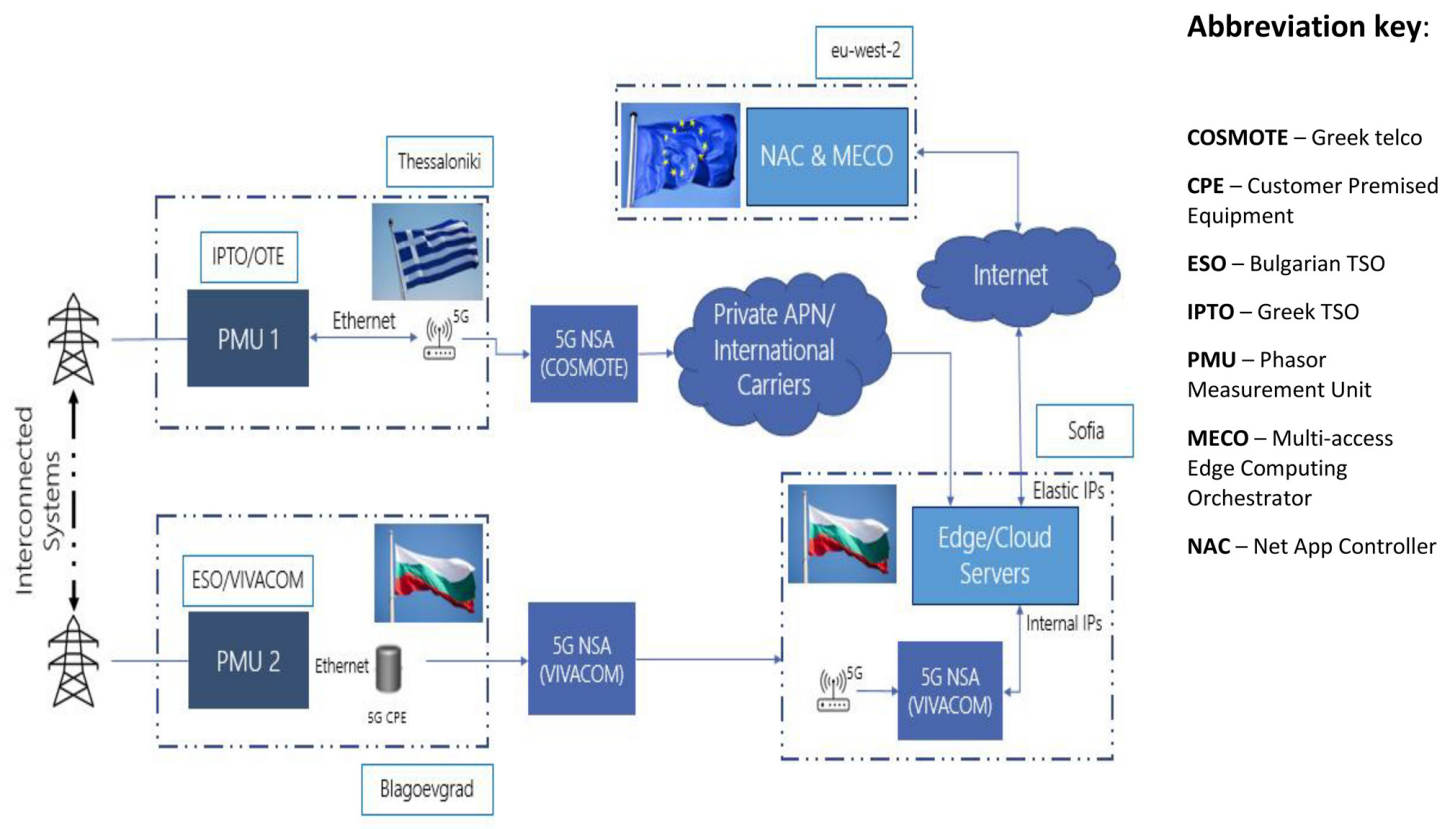
5G data flow of the of UC4 demo (Source: VIVACOM).

**Figure 9.**
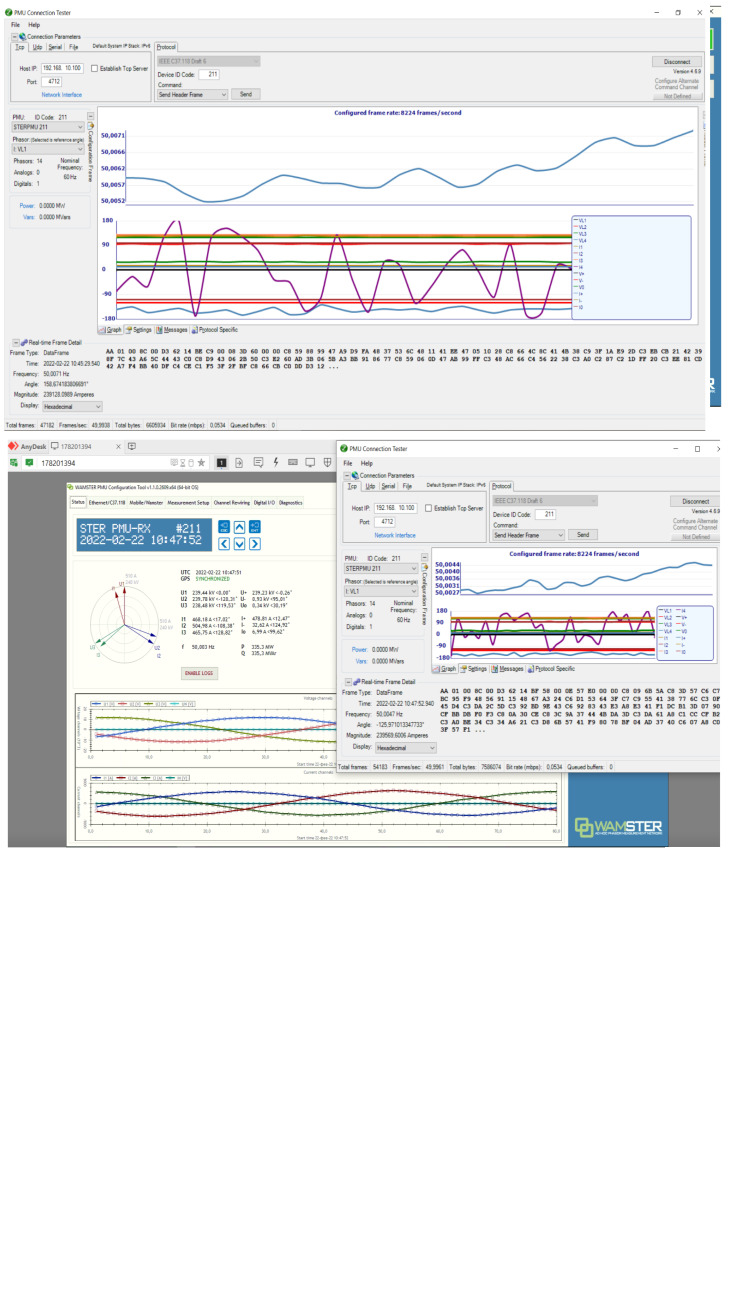
Graphical User Interface (GUI) screenshots of the ESO’s Phasor Management Unit (PMU) with graphs and phasor diagrams.

The 5G data flow diagram for WAM supported by UC4 is illustrated in Figure 8. The left side shows the telco infrastructure starting from the PMUs installed in both Blagoevgrad (BG) and Thessaloniki (GR) substations operated, correspondingly, by ESO and IPTO. The PMU in Blagoevgrad substation transmits measurement packs via 5G CPE (Customer Premises Equipment) through the 5G Non-standalone Network of the Bulgarian telco operator ^28^ to the edge cloud-based vPDC for further processing. At the same time, the PMU in Thessaloniki transfers its measurement packs using the 5G Non-standalone Network of the Greek telco operator. Incoming data from both PMUs are read, synchronized and validated by the edge cloud based vPDC, which then forwards it to the Advisory service whose main function is to provide early warning and suggestions to the TSOs for system security optimisations to improve prevention of critical grid conditions, such as power swings or frequency deviations, that could affect the interconnected power systems. The WAM service supports various PMU status indicators and visualization functions, including a map showing current location of the PMUs; firmware name, address, model, serial number, and version; rated system frequency and actual measured value (frames per second); current and voltage phasor graph with hard real time updates; voltage magnitude and angular difference m based on historical data for both monitored substations. GUI (Graphics User Interface) screenshots of the Blagoevgrad-based PMU (operated by ESO EAD) are shown in Figure 9.

The following PMU GUI screenshots ( Figure 9) illustrate RT measurements of critical parameters that the TSO uses as part of the UC4 demo:

The PMU (GUI screenshot in Figure 9) supports sampling rate of 50 to 60 times per second and includes positive, negative, and zero sequences of voltage (U) and current (I) plus frequency (f), active (P) and reactive (Q) power. It collects this data from the energy infrastructure using measurement inputs ( *e.g.*, from current and voltage transformers and other sensors). Next, all RT PMU measurement data arrives at the WAM and vPDC Services in VIVACOM Cloud. Then, upon validation and synchronization, it goes to the RSC-run Advisory Service NetApp for final processing and advisory to the TSO depending on the type of abnormality found, if any. A non-exhaustive list of advisory services may include, *inter alia*, suggestions to suppress RoCoF deviation, voltage/current phasor abnormalities or power swings, recommendations for activation of Frequency Containment Reserves (FCRs), *etc.*

**Figure 10.**
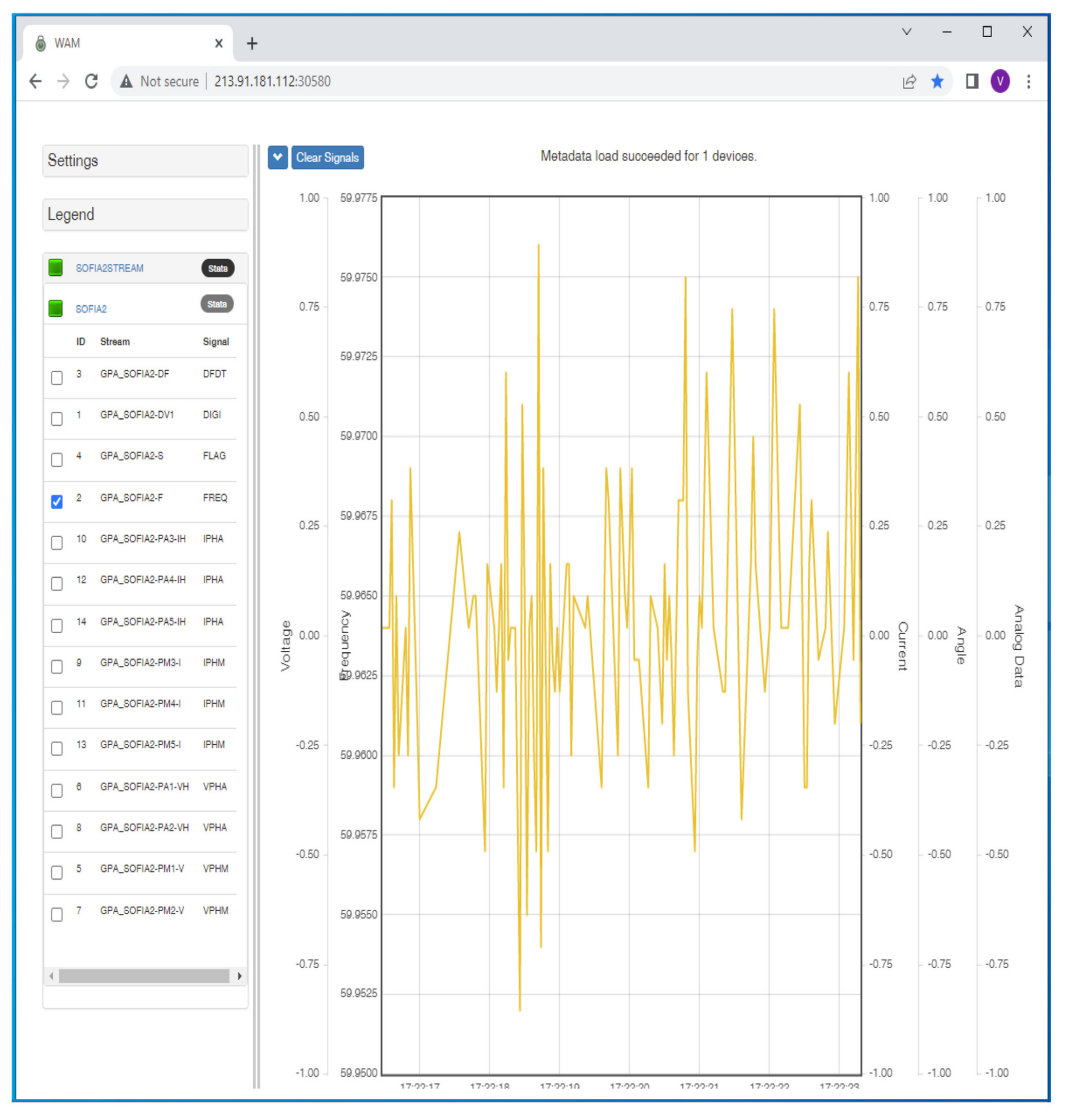
Screenshot of Wide Area Monitoring (WAM) service display.

Figure 10 exhibits a WAM Service metadata display. The instance shown includes four real-time measurement channels: voltage, current, phase angle, and frequency. Derivative products such as P (MW) and Q (Mover) may also be displayed.

### Benefits of 5G Deployment in the Energy Vertical as per UC4 Scenario

**Business:** Optimized power system monitoring by the RSC results in both TSOs having an improved ability to monitor and supervise their control area, being aware of the status of neighboring power grid.

**Economical:** An adequate level of inter-TSO coordination allowing early detection and prevention of potential faults before they escalate into severe contingencies such as outage, brownout, or blackout conditions, thus enabling TSOs to better operate and balance their grids. Moreover, 5G-native improved visibility of critical interconnection grid elements infers higher volumes of cross-border electricity exchange and enhanced market coupling, ultimately generating financial benefits for all involved countries, in this instance Bulgaria and Greece.

**Social**: Secure and uninterrupted power supply to end consumers.

**Environmental:** Higher RES shares in the energy generation mix decrease the use of conventional power plants drastically, leading to massive CO _2_ emission cuts. However, such penetration of renewables also augments system security concerns due to their invertor grid connections and intermittent production patterns. UC4 may hence be viewed as a contributor to enhanced coordination reliability, which is a major prerequisite for future large-scale RES deployment. Therefore, this UC scenario also entails indirect benefits for climate change mitigation benefits.

## Key results and conclusions from TSO’s perspective

The main results reported in this article are the Open 5G Experimentation Platform and the NetApps developed and customized to support the Smart5Grid Use Cases 3 and 4. In specific terms, the NetApps behind UC3 and UC4 are showcased as one of the key pilot results that are illustrative of how these particular results exemplify and demonstrate the Smart5Grid contribution to delivering an added value, flexibility and reliability to legacy wire-bound communication by deployment of 5G network slicing solutions in transmission and distribution grids.

The actually measured E2E latency performance for UC3 is less than 10 msec, which compares to that of fiber optic or wire-bound network while offering additional flexibility in terms of network slicing in the form of URLLC and mMTC. UC3 supports Run Time Energy Production Monitoring and Predictive Maintenance (Enabler) services whose capabilities are demonstrated in the following video file: (download here).

The actually measured E2E latency performance for UC4 is less than 10 msec as well, which compares to that of fiber optic or wire-bound network while offering additional flexibility in terms of network slicing in the form of URLLC and mMTC. UC4 supports Virtual Phasor Data Concentrator, Wide Area Monitoring, and Advisory (Enabler) services whose capabilities are demonstrated in the following video file: (download here).

In practical terms, as intrinsically volatile renewables tend to become dominant DERs in a targeted complete decarbonization environment, power systems face unprecedented security and balancing challenges that need to be adequately addressed by accelerated and cost-effective integration of 5G communication technologies providing TSOs with ultrafast, low-latency and intelligent tools for higher visibility and control maneuverability of rapidly expanding distributed renewable energy resources. In addition to system security benefits at transmission and distribution level, smart grid solutions — as the UC3 pilot demo — could also enable renewable generators to better forecast their maintenance needs and decrease both scheduled and accidental outages and downtime of their assets, leading to operational cost optimisations. This, in turn, also increases renewable generators’ scheduling precision and limit RES curtailment. It thus allow RES (potentially including DRER) to participate in the energy and balancing markets in a more productive manner, offering ancillary services to TSOs; avoiding penalties due to deviations from production schedules; and smoothing out unintentional and excessive power peaks usually seen at change of the hour, especially in intraday market timeframes.

In essence, 5G technology tailored for the energy vertical should not displace traditional FO and PLC (Power Line Communications) assets but rather offer, in a scalable and cost-effective way, critical flexibility, RLLC, mMTC, and eMMB for TSOs to be able to better monitor locations where such 5G energy vertical capabilities are most needed ( *i.e.*, in remote rural areas of massive RES expansion that FO and PLC fail to keep pace with and/or is too costly to build).

In system-wide terms, 5G-supported WAM capabilities as those demonstrated in the Smart5Grid UC4 pilot represent a powerful tool for TSOs to boost their control area monitoring capacity and yield important benefits to legacy fiber optic and wire-bound (PLC) communications commonly used in conventional power grids. Those benefits, as touched upon in the UC4 section, originate from the 5GsmartGrid tools (NetApps) that TSOs can leverage to monitor and control their synchronously interconnected control areas in a more efficient and preemptive manner, based on URLLC and high granularity 5G communications. Such tools allow TSOs to detect and suppress any abnormalities such as frequency deviations or power swings early enough (before their aggravation or escalation into contingencies).

## Disclaimer

The above key observations and conclusions expressed in this article are based on current physical data and results of the Smart5Grid project. Furthermore, as Smart5Grid is a still ongoing Horizon 2020 R&D project, the observations and conclusions contained in this article are neither definitive nor exhaustive but indicative. They may be subject to changes and revisions depending on the final qualitative and quantitative results of this project vs. KPIs.

## Ethics and consent

Ethical approval and consent were not required as part of this study.

## Data Availability

Dryad: Smart5Grid solutions for enhanced TSO grid observability and manageability in massive RES penetration environment. https://doi.org/10.5061/dryad.pvmcvdnq4 ( Shangov *et al.,* 2022) This project contains the following underlying data: UC3_NetApp_Timeseries_of_Realtime_PV_RES_Monitor_Results_1.xlsx UC4_NetApp_vPDC_Timeseries_Results.xlsx Dryad: Smart5Grid solutions for enhanced TSO grid observability and manageability in massive RES penetration environment. https://doi.org/10.5061/dryad.pvmcvdnq4 ( Shangov *et al.,* 2022) This project contains the following extended data: README_Smart5Grid_Article_Sustainable_Places_2022.txt Data are available under the terms of the CC0 1.0 Universal (CC0 1.0) Public Domain Dedication license. Zenodo: Smart5Grid solutions for enhanced TSO grid observability and manageability in massive RES penetration environment. https://doi.org/10.5281/zenodo.7462321 ( Shangov, 2022) This project contains the following extended data: Figure_1.jpg Figure_2.jpg Figure_3a.JPG Figure_3b.JPG Figure_4.jpg Figure_5.jpg Figure_6a.jpg Figure_6b.jpg Figure_7.jpg Figure_8.JPG Figure_9(1).jpg Figure_9(2).jpg Figure_9(3).jpg Figure_10.jpg SP2022_Manuscript_15090_Smart5Grid__Rev_4_Clean.docx Data are available under the terms of the Creative Commons Attribution 4.0 International license (CC-BY 4.0).

## Footnotes

^5^ A Phasor Measurement Unit (PMU) is a devise that measures synchronized magnitude and phase angle values of an electrical phasor variable, *e.g.* current or voltage, in a power grid. Such synchronized measurements are known as synchrophasors. A PMU uses a common source of time synchronization such as Global Navigation Satellite System (GNSS) module/receiver and has wire- bound as well as wireless communication capabilities for data exchange with Supervisory Control And Data Acquisition (SCADA) systems.

^6^ HIL is a testing setup where real signals from a controller are connected to a testbed that mirrors (simulates) a real/physical system that the controller is tricked to consider as such instead of as a digital twin system. This enables running multiple test and experimentation scenarios as though a real system is being used but without the risks, costs and time associated with physical tests.

^7^ A phasor is a complex number representing a sine function whose amplitude (A), angular frequency (ω), and initial phase (θ) are time invariant. A common situation in electrical grids is the existence of multiple sinusoids, all having the same frequency but different amplitudes and phases. The only difference in their analytical representations is the complex amplitude (phasor). A linear combination of such functions can be included in the product of a linear combination of phasors (known as phase arithmetic) and the time/frequency dependent factor that common to all. Hence, a vPDC is a MEC/cloud based device that collects and synchronizes measurements from PMUs before transferring them

^8^ https://github.com/argoproj/argo-workflows

^9^ https://www.keycloak.org

^10^ https://gitlab.com/gitlab-org/gitlab

^11^ https://goharbor.io

^12^ https://github.com/elastic/elasticsearch

^13^ https://github.com/elastic/logstash

^14^ https://github.com/elastic/beats

^15^ https://www.gridprotectionalliance.org

^16^ https://github.com/GridProtectionAlliance/gsf

^17^ https://github.com/GridProtectionAlliance/openPDC

^18^ https://github.com/GridProtectionAlliance/openHistorian

^19^ https://www.enel.it

^20^ https://www.edistribucion.com

^21^ https://www.entra.energy/

^22^ In UC3 demo, the wind farm consists of a single wind turbine with installed capacity of 2MW

^23^ The 5G communication requirements for both UC3 and UC4 are based on the 3GPP TS22.261 and ITU-T G.827 standards

^24^ www.eso.bg

^25^ www.admie.gr/en

^26^ Model and performance of the PMUs used for the UC4 demo: STERPMU-Rx, free standing unit, 32GB local flash memory, embedded STERLINK communication and GNSS module, 3Xu +3XI measurement terminals, GNSS receiver for time synchronization, 1xEthernet 10/100Mbps port, 1x4G modem, configuration tool

^27^ In the context of the bandwidth requirement, Nod refers a PMU connection point with the network.

^28^ https://www.vivacom.bg

## Software availability

The open Smart5Grid Open Experimentation Platform is developed using open-source software available from the following public links: https://github.com/argoproj/argo-workflows; https://www.keycloak.org; https://gitlab.com/gitlab-org/gitlab; https://goharbor.io; https://github.com/elastic/elasticsearch; https://github.com/elastic/logstash; https://github.com/elastic/beats

## References

[ref-5] Shangov D : Smart5Grid solutions for enhanced TSO grid observability and manageability in massive RES penetration environment. Dryad, [Data], 2022. 10.5061/dryad.pvmcvdnq4 PMC10521069 37767203

[ref-6] Shangov D : Smart5Grid solutions for enhanced TSO grid observability and manageability in massive RES penetration environment. *Zenodo* . 2022. 10.5281/zenodo.7462321 PMC10521069 37767203

