# Smart5Grid Solutions for enhanced TSO grid observability and manageability in massive RES penetration environment

**DOI:** 10.12688/openreseurope.15090.2

**Published:** 2023-07-26

**Authors:** Daniel Shangov, Krassimir Vlachkov, Ralitsa Rumenova, Georgi Hristov, Atanas Velkov, Angelos Antonopoulos, Nicola Cadenelli, Nikolaos Tzanis, Dimitrios Brodimas, Michalis Rantopoulos, Ioannis Chochliouros, Vasiliki Vlahodimitropoulou

**Affiliations:** 1 Project Management, Elektroenergien Sistemen Operator (ESO EAD), Sofia, Bulgaria; 2 Entrea Energy, Sofia Tech Park, Bulgaria; 3 Bulgarian Telecommunications Company EAD, Sofia, Bulgaria; 4 Nearby Computing, Barcelona, Spain; 5 IPTO (ADMIE), Athens, Greece; 6 Hellenic Telecommunications Organization S.A. (OTE), Athens, Greece

**Keywords:** Smart Grid, 5G Open Experimentation Platform, Open Service Repository, Verification and Validation, NetApps

## Abstract

This article presents the latency minimisation potential provided by the Smart5Grid Open Experimentation Platform (OEP) developed by the Horizon 2020 Smart5Grid Research and Innovation (R&I) project. It discusses the OEP performance and provides experimental data to substantiate its contribution to improving observability and manageability of distributed renewable generation in power grids. That experimental proof is delivered by two pilots running on the OEP: Demo 1 Millisecond Level Precise Distribution Generation Control, and Demo 2 Real-time Wide Area Monitoring (WAM) pilot of 5G virtual Phasor Data Concentrator v(PDC) capabilities for WAM of end-to-end electricity grids. This work reports  two Network Applications (NetApps) created to support both demos and provides experimental evidence that the OEP offers latency of comparable measure to well-established wire-bound communications in addition to availability and reliability on top of by-design flexibility, scalability and modularity, which are especially relevant to power systems with high shares of Distributed Renewable Energy Recourses (DRERs). The software and methods used for the OEP development and experimental testbeds applied to measure its latency performance in both tailored pilot demos are explained at length. The test results are presented and interpreted with a view to discussing potential contributions of the presented 5G-enabled solutions for power grid smartification in conditions of high rollout of distributed renewable generation. All pilot demos generate openly accessible data, except where specific security restrictions are applicable.

## Related works

Power grid digitalisation implies advanced communication, automation and control [ Pau *et al.*, 2022]. Hence, modularity, scalability, and interoperability need to evolve consistently in smart grids with service-oriented network design [ Pau *et al.*, 2022]. An essential functionality for many smart grid applications is the Wide Area Monitoring, Protection and Control (WAMPC) of interconnected transmission grids [ Asprou *et al.*, 2022]. Such applications rely heavily on time-aligned measurement technology and large-scale deployment of Phasor Measurement Units (PMUs) in power substations. A PMU is an advanced GPS-clock synchronized equipment that provides voltage and current phasor measurements, frequency, and Rate of Change of Frequency (RoCoF = $\frac{df}{dt}$ ) with precision and sampling rates (e.g., 200 phasors per second in a 50 Hz system) that are substantially higher than earlier generation gird measurement equipment (e.g., every 2–5 sec in asynchronous conditions) [ IEEE standard for synchrophasors for power systems, 2011]. Fiber optics (FO) is the well-established wire-bound data carrier connecting filed PMUs with Supervisory Control and Data Acquisition (SCADA) systems. However, while FO supports WAMPC (e.g., securing network delays within 100–150 ms), its rollout cost and impossibility of flexible and fast (re)deployment could be a deterring factor, especially for smart grids where modularity, scalability, and flexibility are key. On the other hand, PMU deployment in power grids has not yet become commonplace, with many substations still using asynchronous measurement assets [ Sood *et al.*, 2009]. Against this backdrop, DRERs tend to spread massively to support the energy transition, which requires that the interaction between SCADAs and field devices, such as PMUs, be based on a smart network service architecture with Ultra Reliable Low Latency Communication (URLLC) and response-time optimisation capability. The knowledge field of exploration this work has attempted to address is the latency optimisation potential of 5G-based flexible, scalable and reproducible solutions for WAMPC of interconnected power systems as well as for millisecond precise monitoring of distributed renewable generation assets.

## Introduction

This work demonstrates the latency optimisation capacity of the 5G-enabled Open Experimentation Platform (OEP) developed by the Smart5Grid project by providing experimental evidence of its service slicing in the form of URLLC tested validated in two demos that could guarantee the required Quality of Service (QoS) standards for energy vertical communication networks. Specifically, a 5G network has a packet loss rate in the range of 10 ^-5^ and theoretical latency in the Radio Access Network (RAN) side of 1–3 ms [ 5G PPP, 2014]. It has recently been acknoledged that such features are of high relevance for Wide Area Monitoring, Protection and Control (WAMPC) in order to enable timely response to system faults or contingencies [ 5G PPP, 2014]. This paper substantiates the OEP performance allowing power grids to have higher observability and manageability over Distributed Renewable Energy Resources (DRER), in both internal and interconnected power system level. It addresses leveraging URLLC data exchange via the 5G-enabled OEP for precise real-time Wide Area and Monitoring (WAM) using measurements to counter preemptively inter-area frequency oscillations and power swings [ Khan & Yan], boosting effective maintenance of grid stability and reliability. It also demonstrates a 5G-enabled run-time millisecond precise distributed renewable generation monitoring solution and provides experimental evidence of its latency performance. Finally, key observations and conclusions are drawn on the potential implications and possible future explorations in the domain of data exchange optimisation in critical energy vertical systems such as power grids.

## Methodology

### Elaboration of the 5G Open Experimentation Platform and Demos

The 5G Open Experimentation Platform (OEP) development methodology is based on the concept of Network Applications (NetApps). Their main objective is to hide the complexity of a 5G telco network for energy application developers in a way that empowers them to develop a NetApp without having to deal with the underlying network. A Virtual Infrastructure Manager (VIM) such as OpenStack or Kubernetes hosts every unit that composes a NetApp. The VIM provides monitoring data to a Network Function Virtualisation Manager and Orchestrator (NFV MANO) framework, which airs information to the NetApp Controller (NAC) that employs analysis algorithms to propose the optimal Virtual Network Function (VNF) [ ETSI, 2018] and NetApp placing. A Slice Manager (SM) reserves resources for all these capabilities. Adaptable architectural design builds an integrated infrastructure accommodating the entire spectrum of energy vertical’s communications and computational needs. The OEP Verification and Validation (V&V) framework is developed using API Handler based on Golang gin-gonic framework, its Pipeline engine is based on Argo Workflow ^1^, and its Results Manager uses Golang with MongoDB. The Open Repository (OSR) Authentication and Authorization Service is created using Python programming language, and the driver software implementation is based on Keycloak ^2^ open-source software for user management and single-sign-on (based on OpenID connect) for all OSR components and the OEP User Interface. Python programming language is also used for the OSR Authentication and Authorization Service and driver software implementation.

Keycloak open-source software is employed for user management and single-sign-on (based on OpenID connect) for all OSR components and the OEP User Interface. The OSR NetApp Catalogue Programming Code Language (and framework) is Python (Django), Database: PostgreSQL. The OSR Code Versioning Service is created using Python programming language for the driver software implementation Integration with GitLab ^3^ open-source software to store object descriptors and keep track of their different versions. The OSR Images Registries are likewise based on Python programming language for implemented driver software Integration with Harbor ^4^ open-source software to store container images and Helm charts. The OSR Event Logging Service is heavily reliant on Elasticsearch ^5^, Logstash ^6^, and Beat agents ^7^ open-source software. Elasticsearch is used as a distributed log database and search engine while Logstash modifies logs to a unified format Beat agents in each logged component to gather and push logs to Logstash.

Using that reference design, Smart5Grid has created an integrated Development and Operations (DevOps) methodology manifested as a Verification and Validation (V&V) framework of NetApps and Network Services (NSs) as Virtual Network Functions (VNF) graphs enabling operators to monitor their behaviour.

**To validate the OEP’s latency performance, two demos are explored in this paper**: Demo 1 of Run-Time Energy Production Monitoring and Predictive Maintenance (Enabler), and Demo 2 of virtual Phasor Data Concentrator (vPDC), Wide Area Monitoring and Advisory Enabler (WAM&A), both developed by means of Grid Protection Alliance (GPA) ^8^ open source software and GSF open source library ^9^. Another two GPA open source projects used for both demos development are openPDC ^10^ and openHistorian ^11^. Microsoft Visual Studio 2022 is the coding environment while C# и JavaScript are the programming languages engaged in the demos NetApp development process. Correspondingly, the services for both demos are placed in Docker images that are used in Helm charts. These Helm charts serve to install the services in Kubernetes cluster, which runs Docker containers of the services in its cluster. Detailed video demonstrations of both use cases’ services are referred to in the results section of this paper. 

A digital twin and Real Time Hardware-In-the-Loop (RT-HIL) pre-piloting testing is applied as an advanced analytical, trial and data collection method to simulate and investigate power system phenomena and components in both Demos. Realistic and flexible experimentation and testing conditions for de-risking equipment are its key benefits. This methodology creates a common basis for testing and facilitating the analysis of renewable energy integration at power distribution and transmission levels.

### Experimental setup and data collection methods

**Figure 1.**
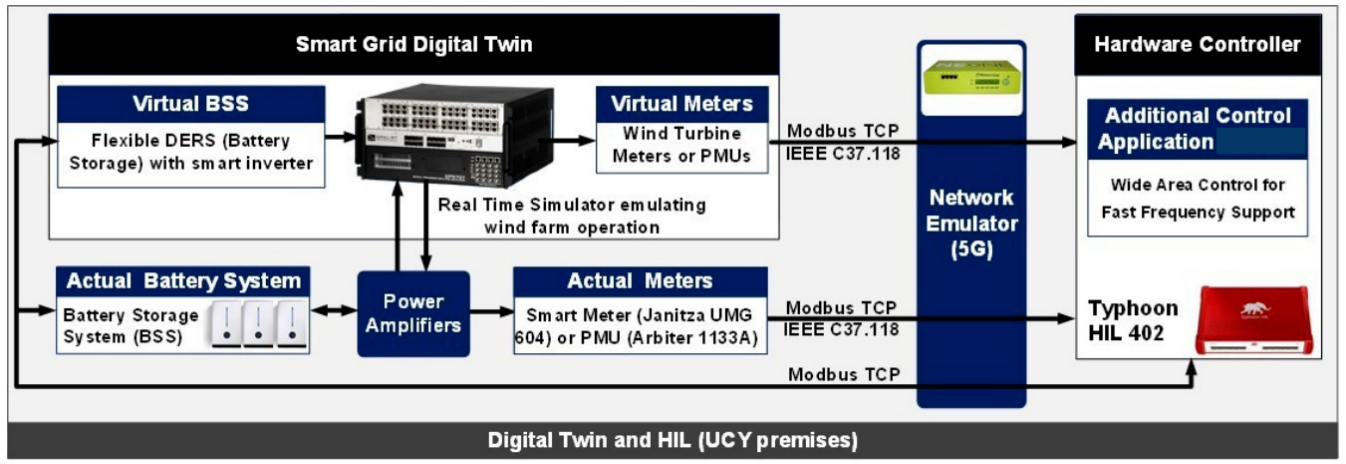
RT-HIL testbed for Demo 1.

**The Demo 1 RT-HIL testbed** as evident in Figure 1 is committed to integrating and validating the millisecond level precise distributed generation functional requirements. Its core component is an Internet-of-Things (IoT) device (Raspberry Pi 4 Computer Model B; BCM2711 SoC; 4GB DDR4 RAM; USB 3.0; PoE Enabled) that reads a signal list form SCADA and sensors installed on the wind turbine by OPC client-server protocol. The IoT device is equipped with 5G HAT installed with 5G SIM that acts as a 5G gateway channeling the signals via private Access Point Name (APN) gateway to the MEC server of Vivacom lab on top a of public 5G Non Standalone network. To complement Demo 1, two control applications were developed: (1) Fast Frequency (FF) support, and (2) Ramp-Rate (RR) limitation services. These were tested using the Real-Time Hardware-In-the-Loop (RT-HIL) method. They demonstrate the potential of 5G-communication for enhancing the eligibility of such units to provide flexibility services in real-time electricity markets using a non-invasive and realistic operational environment of the grid. The test setup for the FF controller is shown in Figure 1. The RR service setup is designed likewise. Its main component is the RT-HIL OPAL-RT OP5707 real-time simulator. It receives run-time measurements from actual battery storage and electrical, mechanical and environmental meters installed on a real 2MW wind turbine to create a digital twin that consists of of a virtual wind turbine with mirrored metrics as well as a virtual battery storage system. 

**The method of data collection and exchange** between actual and virtual testbed components uses IEEE C37.118 Modbus Traffic Control Protocol. A lab-based network emulator employing Typhoon HIL 402 technology is the device that serves to test the network behaviour of both control applications in order to verify their 5G-enabled latency performance. 

**Figure 2.**
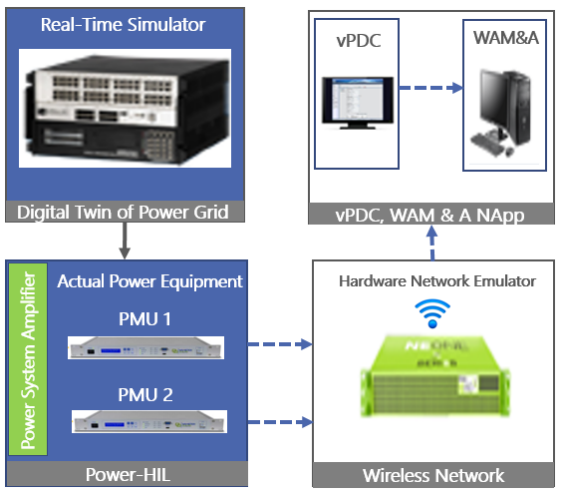
RT-HIL testbed for Demo 2.

**The Demo 2 RT-HIL testbed** for integration and validation of the WAM and Advisory (WAM&A) NetApp functional requirements is shown in Figure 2. Its core also includes RT-HIL OPAL-RT OP5707 real time simulator, i.e. a hardware network emulator that creates a digital twin of the tested real grid element (400 kV interconnector between Bulgaria and Greece), and two actual PMUs connected in a power-HIL setup. The two PMUs are installed at the opposite end substations of the existent 400 kV interconnector between Bulgaria and Greece. A docker image of the WAM and WAM&A is installed locally in the testbed. Description of each testbed component and the digital twin models of an IEEE 9-bus test system used to investigate the impact of communication layer on the WAM&A service is available in [ Asprou *et al.*, 2022]. The actual NetApp (local docker file) is integrated in the RT-HIL testbed.

The WAM&A NetApp was installed on a cluster of edge cloud servers (in Vivacom labs) managed by Kubernetes, where the Docker images of the services were located in Docker Hub repository. From that repository, the WAM&A service is accessible to the RT-HIL testbed via web browser on a dedicated port (e.g., port 1010, thus http://localhost:1010/), using preset credentials. In the RT-HIL testbed, a Virtual Phasor Data Concentrator (vPDC) ^12^ receives phasor readings from both PMUs and delivers a data stream with time-aligned and stamped measurements to the WAM&A service. To visualize the interconnector run-time condition, WAM&A obtains a time-aligned PMU stream (known as synchrophasors) from the vPDC and produces graphs for grid operators, which obtain the current phasor that flows through the interconnector and the voltage phasors from the opposite ends. In the case of Advisory service, synchronised measurements received from vPDC are stored in a database and used to produce statistical analysis for the grid operator. Two relative case studies were run to verify that the NetApp adequately captures and monitors all operational conditions: (1) normal load and (2) transients (fault event).

The latency test results for both demos reported and discussed further in this paper were generated by series of ping tests and ZABBIX network monitoring tools performed at the edge cloud (MEC) servers hosting the NetApps. The response time statistics were recorded in several sequences of 7-day continuous measurements. A Non-Standalone (NSA) 5G public network with dedicated APN and SIM card was leveraged for the field deployment of both pilot demos. The underlying 5G NSA architecture is displayed and described in the “Pilot demos explored” section of this works.

### The underlying 5G-enabled Open Experimentation Platform

**Figure 3.**
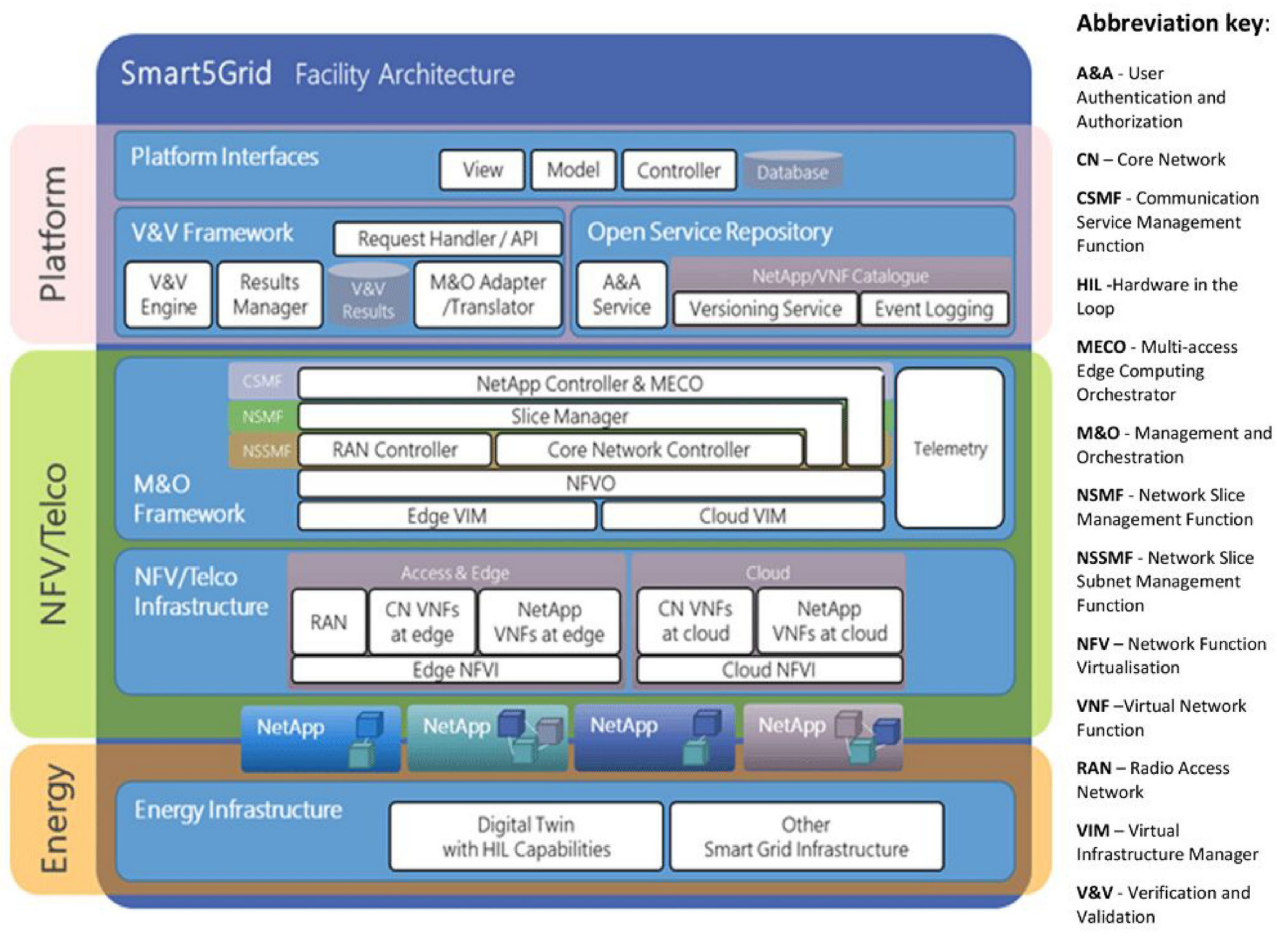
Overall design of the Smart5Grid Open Experimentation Platform (OEP).

The main product of the Smart5Grid project is the **5G Open Experimentation Platform (OEP)** ( Figure 3) enabling stakeholders in the energy vertical, ICT integrators, Network NetApp) developers, telecom industry actors, SMEs, and/or network service providers in general to test, validate and share/expose their NetApps and create 5G open-source repositories for wide use and supporting standardisation.

### The Smart5Grid Open Experimentation Platform

The **OEP** is designed to support applying and scaling-up Multi-Access Edge Computing (MEC) across the energy vertical, with particular focus on power grids. The main objective is to bring computation, storage, and network resources “closer” to the devices that make up power grids in order to solve inherent resource limitation issues and offload NetApps directly to MEC servers. This aims to transpire into substantial reduction of End-To-End (E2E) latency of devices accessing telco networks and, correspondingly, significant energy savings. MEC supports data security and integrity as well by supporting ubiquitous last-mile service access to smart grid devices, while offering ultrafast and reliable deployment of network slices and value-added capabilities for the smart grid NetApps, such as bandwidth assurance, life cycles management of network services, and overall balancing of service loads.

As illustrated in Figure 3, the **Smart5Grid infrastructure consists of** three main interdependent and interfaced layers: (i) **Energy Infrastructure**; (ii) **Network Function Virtualization (NFV)/Telco**, and (iii) **the Open Experimentation Platform on top**. The platform itself is composed of **Open Service Repository (OSR), Verification and Validation (V&V) Framework, the NetApp Controller (NAC)**, and dedicated Application Programming Interfaces (APIs) and User Interfaces (UIs). The V&V Framework includes Verification and Validation (V&V) Engine, Results Manager, V&V Results container, and Management & Orchestration (M&O) Adapter and Translator. The OSR consists of an Authentication & Authorization (A&A) Service and NetApp/VNF Catalogue with Versioning Service and Event Logging components. This Catalogue serves to store ready (meaning verified and validated) NetApps that can be used by developers, for instance to assemble new NetApps. It is the OSR where NetApps are managed ( *i.e.*, upload, download, edit, delete, shared, *etc.*) following user identification (ID) and access routines. The V&V is where they undergo testing, verification, and validation prior to going live. As a key component of the platform, the NAC is architecturally located on system management level and hosts the MEC offloading and Elastic VNF sizing and chaining functions. Its main responsibility is to save energy, optimize and accelerate data processing by proper parallelization and code partitioning of NetApps offloaded between the centralized platform and its MEC counterpart. Since latency minimisation is one of the key objectives of Smart5Grid, it is met by one-dimensional search algorithm that follows an optimal offloading decision policy according to the application buffer queuing state, available processing powers of MEC servers, and characteristics of the Channel States through Channel State Information (CSI) between the MEC servers and the Phasor Measurement Units (PMUs) ^13^ and other metering devices.

The energy infrastructure representing the platform’s bottom hierarchy layer in Figure 3 includes a digital twin of (part of) the power grid used for Hardware-in-the-loop (HIL) ^14^ experimentation and testing with RT data according to the concrete pilot setup, *e.g.*, deriving from PMUs and virtual Phasor Data Concentrators (vPDC) ^12^ . It also accommodates other smart grid hardware such as Intelligent Electronic Devices (IEDs), metering equipment, sensors, *etc.* This is where NetApps interface with the NFV/Telco layer consisting of Edge and Cloud Network Function Virtualization Infrastructure (NFVI), in turn composed of Radio Access Network (RAN) as well as edge and cloud Core Network (CN) and NetApp Virtual Network Functions (VNFs). The NFV/Telco layer itself is subordinated to the Management and Orchestration Framework (M&O) with its Communication Service Management Function (CSMF), Network Slice Management Function (NSMF), and Network Slice Subnet Management Function (NSSNF). These functions support, correspondingly, the NetApp Controller & Multi-access Edge Computing Orchestrator (MECO), the Slice Manager, and the RAN and CN Controllers interacting via Telemetry (data buses) with the Network Function Virtualization Orchestrator (NFVO) and the Virtual Machines (VIMs) of cloud and edge resources.

### Pilot demos explored

This work discusses Demo 1 of Millisecond level precise distribution generation control (Bulgarian demo) to demonstrate precise monitoring of distributed generation at the millisecond level, which addresses the field of operation and maintenance of distributed power generation with a specific emphasis on renewable sources. It also explores Demo 2 **of** Real-time wide area monitoring (Bulgarian-Greek demo) to showcase a 5G solution for Wide Area Monitoring (WAM) of large interconnected power grids. For this purpose, 5G MEC vPDC capabilities were used for experimentation WAM of end-to-end electricity grids through inter-Transmission System Operator (TSO) Regional Security Coordination (RCS).

### 5G-enabled demos for enhanced observability and manageability of massive RES penetration

The two demos subject to this work demonstrate how the OEP Network 5G slicing capabilities such as URLLC (Ultra-Reliable and Low Latency Communications) could provide TSOs with a high level of observability and manageability of Distributed Renewable Energy Recourses (DRERs). The focus is on technical (frequency response, voltage and load flow control) as well as market (electricity and balancing) implications of high-speed flexible 5G communications for power grids.

### Demo 1: Millisecond level precise distributed generation monitoring

***Description and overall context.*** This experimentation demo is precise monitoring of DRERs at millisecond level, which addresses the field of operation and maintenance of distributed power generation with a particular emphasis on renewable sources. More specifically, real-time (also referred to as or run-time) (RT) monitoring of a wind farm, situated in southeast Bulgaria (Sliven area) and owned by Entra Energy ^15^, is carried out using 5G wireless networks. RT monitoring is vital for the proper operation of wind farms for two key reasons: (i) a RES owner, being aware of the real-time status of its power asset, is able to predict and prevent in time potential future malfunctions that would cause significant financial losses; (ii) RES owners acting simultaneously as BRP (Balancing Responsible Party) and BSP (Balancing Service Provider) are responsible for potential imbalances and for providing real-time balancing services to the market. Precise RT high-granularity monitoring of electricity generation enables DRER owners to minimize their costs while meeting the standardized conditions for providing additional flexibility services (voltage, power, frequency and generation control, *etc.*) through flexible plant management.

Stringent requirements set by TSOs for the provision of RES services make it incumbent on renewable asset operators to use a highly reliable and secure communication between physical assets (in this instance, a wind farm) and TSO dispatch center (SCADA). By engaging millisecond data exchange and native scalability of 5G technology, this can facilitate generation forecast for balancing purposes, optimize energy costs, and visualize end users’ behavior in order to optimally manage their energy profile and provide flexibility services to electricity markets (intraday and balancing market) for multiple in nature (wind, solar, hydro) and geographically spread RES producers.

The following example illustrates potential benefits in terms of operational availability: wind farm (RES) owners or/and TSO are interested in being confident that the RES asset is currently in operation. Occasionally, maintenance, inspection, or unforeseen events may disrupt power plant operation. In such cases, should the plant manager omit notifying the TSO, this may lead to high balancing costs and, correspondingly, unbalanced penalties. RT monitoring can detect such errors in time, preventing escalation and allowing cost optimisation. As for flexibility service provision, the wind farm can deliver its operational data in real time to system operators (TSOs/DSOs), which enables flexible plant management with precise and reliable frequency and voltage control services by the TSO. It should be noted in this context that large windfarms have a communication line with the TSO’s SCADA, usually power line carrier or FO. This is not the case for smaller, distributed windfarms such as the one we used in the Millisecond level precise distributed generation monitoring demo. It is not currently incumbent on small windfarms to meet the same obligations as large windfarms. Nevertheless, this might and is likely to change in the future so that all distributed generation, irrespective of their size, are allowed to participate in the balancing market. In either instance, 5G offers flexibility and scalability at lower costs compared to FO, especially when it comes to remote locations where such communication infrastructure (FO) is either not available in a reasonable proximity or is not feasible to deploy fast enough to accommodate RES penetration.

**Figure 4.**
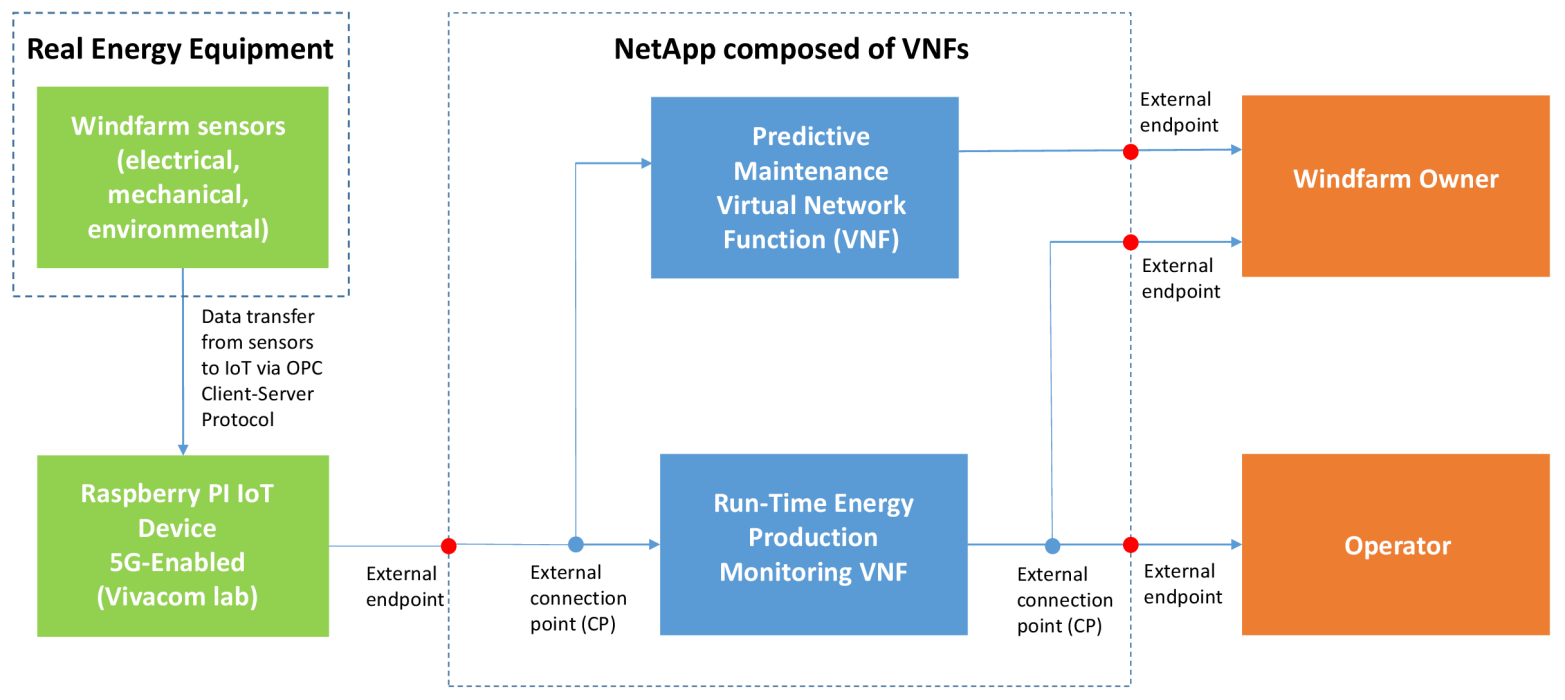
Demo 1 NetApp architecture.

***NetApps supporting the Demo 1.*** There are two services (referred to as Virtual Network Functions, VNFs) supported by one tailored NetApp whose architecture is illustrated in Figure 4: i) Predictive Maintenance Enabler (VNF1 based on massive Machine Type Communication, mMTC) and ii) Run-Time Energy Production Monitoring (VNF2 based on URLLC). Each of these two components is implemented as a different VNF.

The preventive maintenance enabler (VNF1) has two external Connection Points (CPs), one being data input located in the telco (Vivacom) cloud to collect data from the Raspberry Pi device (Raspberry Pi 4 Computer Model B; BCM2711 SoC; 4GB DDR4 RAM; USB 3.0; PoE Enabled) set up to support 5G connectivity (5GHAT module), whilst the other one functions as data output transferring the result of internal processes to the farm owner (or to a 3 ^rd^ party maintenance service provider in the future). The real-time operation functionality (VNF2) also has two external CPs, one being data input located in the telco (Vivacom) cloud to collect data from the Raspberry Pi IoT device, whilst the other functions as data output transferring RT electricity generation signals to both the wind farm owner and the TSO. Both components feature Connection Points (CPs) to render access for setup or troubleshooting purposes.

The two specific VNFs of the Demo 2 NetApp operate as follows:

**VNF1 (Maintenance)** collects measurements from sensors of electrical, mechanical and environmental variables. Electrical metrics include turbine output voltage, current, active and reactive power, and frequency. Mechanical metrics include rotor speed, vibration, operating temperature as well as other variables. Environmental metrics comprise ambient temperature, wind speed, solar radiation, and humidity. The measurement sensors are installed on a 2MW wind turbine capturing the RT performance of key wind turbine components ( *e.g.*, generator, transformer, invertor, 20 kV switchgear). **VNF1** offers to the plant owner information on its generation parameters and serves as maintenance recommendations enabler by providing: (i) RT system alarms information to the asset owner, as well as; (ii) it serves as an enabler for building a NetAapp add-ons that will allow the 3 ^rd^ party providers ( *e.g.*, OEM-Original Equipment Manufacturers or Maintenance Service Providers) to leverage on this RT data and develop predictive maintenance algorithms and functionalities as part of the experimentation and post project market development phase.

**VNF2 (Monitoring)** provides RT data monitoring (in milliseconds from instrument transformer output) of wind farm ^16^ production to the power system owner and operator ( *i.e.*, TSO, in this specific instance the Bulgarian ESO EAD). By knowing production on a millisecond basis, TSOs are able to minimize total system costs while wind farm owners can benefit financially by providing innovative services to system operators (transmission system operator, distribution system operator or both depending on whose grid the wind farm is connected to), such as voltage and frequency control. In addition, it also enables transfer of RT operational data to grid operators, informing them about the wind plant availability in real-time. In essence, both plant owners and grid operators monitor RES asset’s real-time generation in millisecond resolution. By using NetApp 1 capabilities, the wind farm owner can increase the efficiency and accuracy of generation control, forecasting and scheduling also using other information, such as weather data. On the other hand, the grid operators can improve power system stability by monitoring RES output in hard real time.

**Figure 5.**
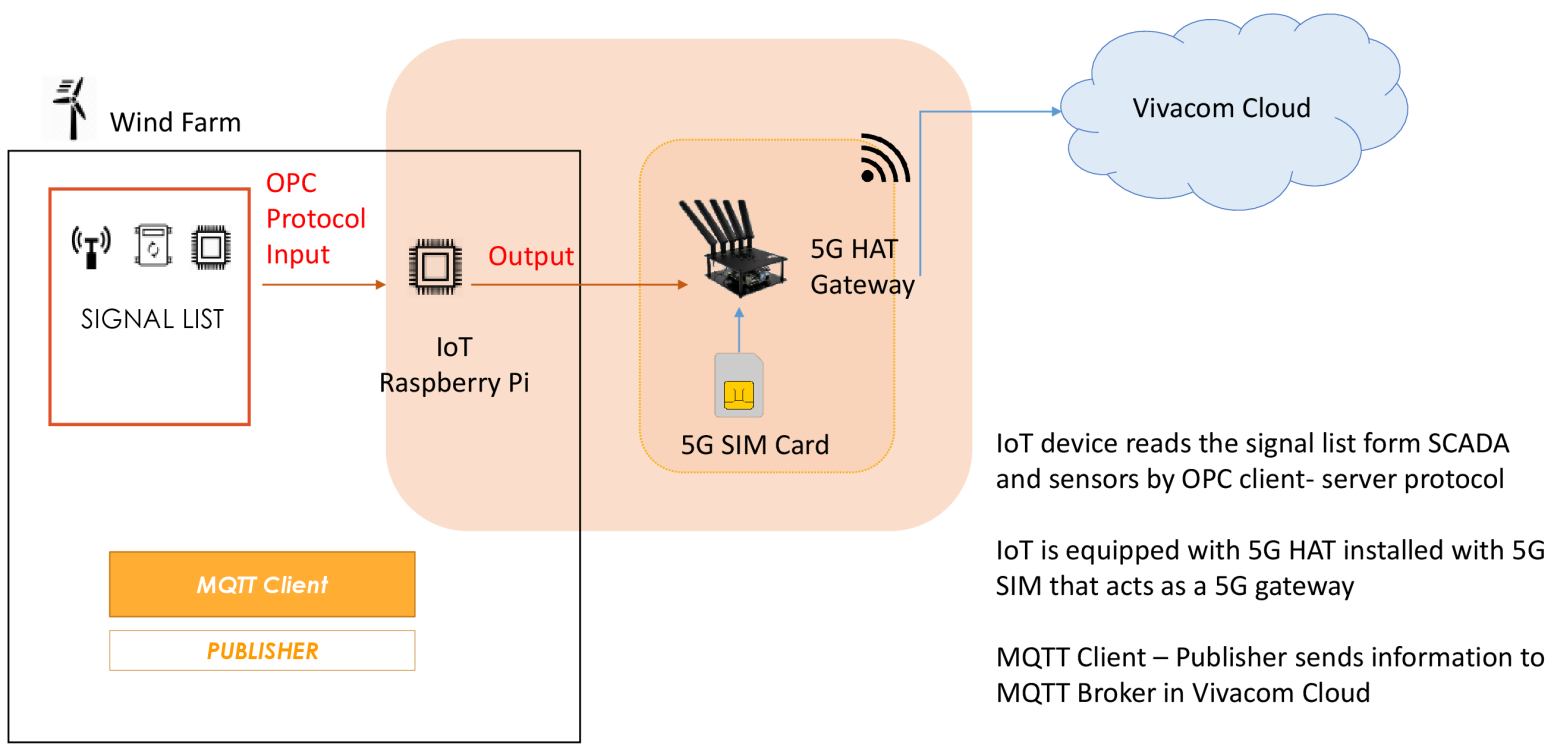
Demo 1 E2E Data Flow diagram with MQTT protocol for data transfer between wind turbine and NetApp.

Figure 5 illustrates the data flow between the wind farm’s Internet of Things (IoT) monitoring sensors and the NetApp functionalities using cloud-based Message Queuing Telemetry Transport (MQTT) Broker/Client servers on top of an Access Point Name (APN)/IP Network Non Standalone 5G architecture of Vivacom. An array of electrical, mechanical and environmental sensors are installed on a real wind turbine and feed RT measurement data to wind turbine owner’s local Supervisory Control and Data Acquisition (SCADA). A 5G HAT equipped Raspberry Pi4 reads those RT measurements (signal list) from the sensors and SCADA using OPC Client-Server protocol and broadcasts them through the 5G HAT gateway with 5G SIM to the Vivacom Cloud. In this data flow configuration, MQTT Client – Publisher transfers signal list data to MQTT Broker in the Vivacom Cloud from where the NetApps serve the Customers (RES asset owner and grid operator) by delivering the demo run-time millisecond monitoring functionalities. Demanding requirements for availability and reliability of communication services (as specified below for the demo) are met through adequate resilience and redundancy measures. This approach enables easy scalability across different types of RES producers (wind, hydro and solar) thus allowing the input from different type of sensors or plant SCADA systems to get connected and emulated using 5G IoT infrastructure in a reliable and secure manner.

RT measurements are communicated using the 5G infrastructure illustrated in Figure 5 to ensure reliable and secure operation of the wind farm and strengthen its Balancing Responsible Party (BRP) and Balancing Service Provider (BSP) capabilities. In a general perspective and taking into consideration the scalability potential, such solution could generate benefits to TSOs in terms of higher visibility, predictability and controllability of renewable assets resulting in improved system balancing and security via ancillary services provided by RES owners.

**Main actors and their roles in Demo 1**: TSO in charge of system balancing and procurement of ancillary services; windfarm owner; BSP tasked to deliver balancing services; BRP responsible for preventing imbalances in real-time market segment; Dispatching Center operating the power grid; IoT devises that measure RT operation of different windfarms; UI display visualizing data according to user requirements; Telecommunication Provider that owns, operates and maintains the public Non Standalone (NSA) 5G network and its main responsibility is to ensure stable and reliable 5G coverage meeting the network specifications under the Service Level Agreement (SLA); Infrastructure Owner providing the server that hosts the OEP as well as the NetApps running at the edge.

**Demo scenarios (operational algorithm)** for the energy vertical service delivered by the **Demo 1 NetApp**:

- No abnormalities in wind turbine operation detected, no alerts sent to windfarm operator.
- Identified potential deviation of windfarm operation in proximate future, maintenance projection sent to windfarm owner (in case of predictive maintenance add-on is developed in the post project market implementation phase).
- Run-time windfarm availability disruption (outage) detected, warning sent to both grid operator and windfarm owner.
- Run-time wind generation does not create system stability problem for grid operator, no curtailment needed.
- Run-time wind generation causes system stability problem, grid operator effects curtailment.

**5G network requirements for Demo 1 ^17^:** Ultra Reliable Low Latency Communication (URLLC) service availability $\left(\frac{Tavailable*100\;,\%}{Ttotal}\right)$ and reliability $\left(\frac{Treliable*100\;,\%}{Ttotal}\right)$ of 99.999%, massive Machine Type Communication (mMTC) service availability and reliability of 99%, URLLC E2E latency of 20–200 ms, mMTC E2E latency not critical.

**Key Performance Indicators (KPIs) for Demo 1**: E2E Latency (between user service and endpoints 20–200 ms); reliability; average service creation time cycle reduced to 90 minutes.

### Demo 2: Real-time Wide Area Monitoring

***Description and overall context.*** Demo 2 is meant for real-time monitoring of a wide geographic area of synchronously interconnected power systems. It leverages Vivacom 5G NSA infrastructure to monitor electricity flows on existent 400 kV interconnector between Blagoevgrad (Bulgaria) and Thessaloniki (Greece) substations, focusing on system security coordination by the Regional Security Coordinator (RSC) in Thessaloniki, Greece. Both Transmission System Operators (TSOs) participating in this demo, *i.e.*, ESO EAD ^18^ and IPTO ^19^, are owners of the Southeast Europe Regional Security Coordination Center (SEE RSC) based in Thessaloniki, Greece. RSC is considered as the WAM owner. Real-time power flow monitoring is indispensable for interconnected power system performance optimisation. In this context, Demo 2 is developed to demonstrate SEE RSC live monitoring optimisation. This process uses PMUs located in Southern Bulgaria and Northern Greece to provide inputs (voltage, current and phase angle measurements) to an edge cloud-based (MEC server in telco data center) vPDC. In addition to collecting PMU measurements, the vPDC's role is to ensure that this data is synchronized and validated before being transferred to the SEE RSC. Such time aligned PMU measurements provide high data granularity (sampling rate of 50 to 60 times per second, including positive, negative, and zero sequences of voltage and current). The use of 5G-enabled data infrastructure of this demo provides URLLC connectivity between the PMUs and the vPDC, conforming to strict network requirements, given its criticality for early detection of abnormalities such as power swings or frequency deviations and, consequently, enabling timely countermeasures to avoid disturbances and contingencies. The PMUs, vPDC and SEE RSC arrangement forms the WAM system of this demo.

As DRERs expand at a high pace across Europe, the interconnected synchronous power systems become more complex and difficult to operate. With growing DRER penetration, inverter-connected devices gain on dominance, which results in lacking physical inertia. This, in turn, causes significant fluctuations in the Rate of Change of Frequency (RoCoF), leading to fundamental changes in system dynamics but also affecting electricity and balancing markets [ ENTSO-E]. It is therefore essential to have a WAM system capable of detecting and suppressing dynamic phenomena that create dangerous conditions for the stability of the entire synchronous European power system. Local disturbances can cause system-wide instability [ ENTSO-E]. WAM mainly benefits from the high PMU accuracy and the low latency of 5G mobile networks [ IFIPAICT]. The European synchronous power system has multiple control areas where each TSO is responsible for its own system control. For proper coordination between adjacent control areas, Regional Security Coordinators (RSCs) owned by neighbor TSOs are established. One of the five critical objectives of an RSC is coordinated security analysis across multiple timeframes (day ahead, intraday, and real time). For RT monitoring of their area (including areas controlled by multiple TSOs), RSCs provide advisory services to TSOs to facilitate disturbance-free system operation. In addition, RSCs offer ex-post information (following major network disturbances or frequency deviations) to the relevant TSOs to develop and improve guidance for this type of problem situation. In the context of this demo, the RSC real-time monitoring function is demonstrated with PMU measurements from Southern Bulgaria and Northern Greece monitoring the interconnection area. The involved TSOs ( *i.e.*, ESO of Bulgaria and IPTO of Greece) then use the information about their connected assets and the recommendations from the RSC to better control and suppress events that could jeopardize system stability.

**Figure 6.**
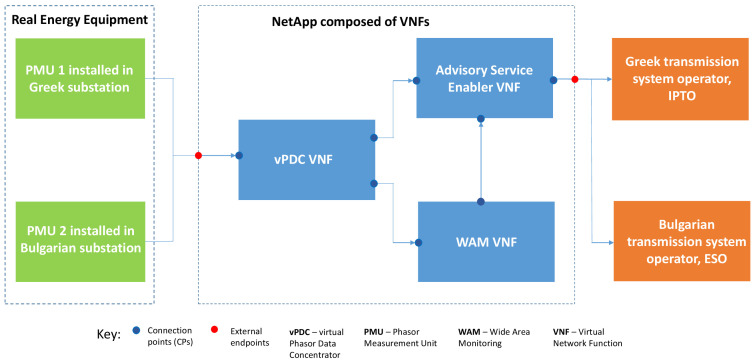
Demo 2 NetApp architecture.

***NetApp supporting Demo 2.*** The Demo 2 NetApp consists of three components (VNFs) whose architecture is illustrated in Figure 6, that is: vPDC, WAM Service, and Advisory Enabler. Each of these components is implemented as a different VNF that is linked and interacts with the other VNFs. Details on their design and creation methodology are provided in the OEP and demo development section of this paper.

**Figure 7.**
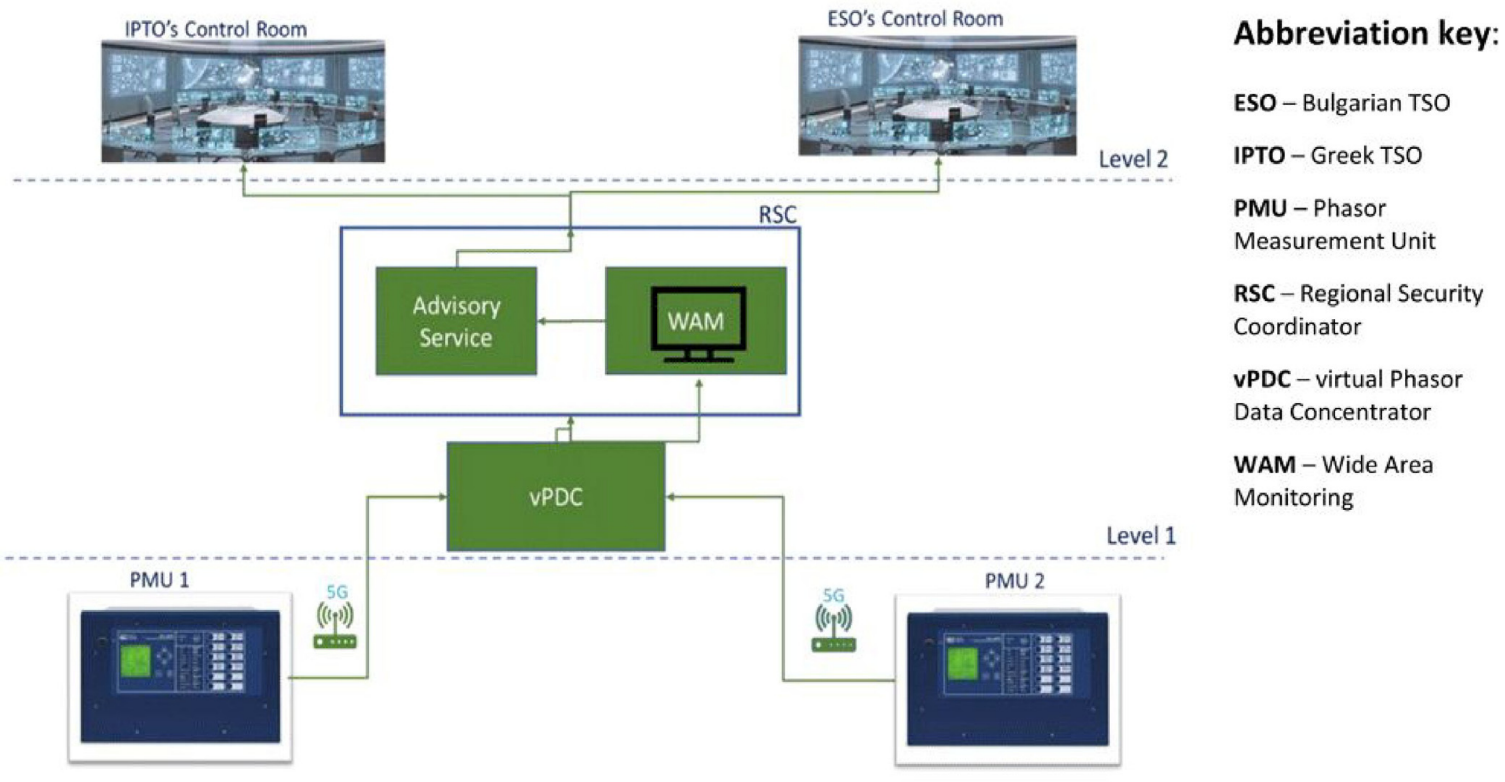
Demo 2 high-level diagram.

Figure 7 illustrates the high-level diagram of demo 2 highlighting its key components and interactions.

First, the vPDC VNF has two Connection Points (CPs) for input from two PMUs located at the opposite interconnector ends ^20^. Assuming the voltages measured by the PMUs at the opposite interconnector ends are V1 and V2, the active power is P, the interconnector reactance is X, and the phase angles of these two voltages are φ1 and φ2, then WAM service determines the power angle δ and the active power as follows:


δ=φ2−φ1(1)

$$P=\frac{V1\;.\;V2\;.\sin\;\text{δ}}{X}\;(2)$$

As δ is the angle that the V1 leads V2, it is a fundamental input for power swing determination, which is a key variable for WAMC of power systems [ Khan & Yan].

Then, after data processing, the output goes to the next components *via* internal CPs. Next, the Monitoring VNF features an internal CP collecting the input from the vPDC, plus one CP to communicate with the Advisory VNF. Finally, the Advisory VNF has two internal CPs for input from the other two VNFs and one external CP to transfer advisory messages to both TSOs (ESO and IPTO).

All three NetApp components have control CPs to render access for setup and troubleshooting purposes. The Monitoring VNF’s control CP will also be used for the RSC (NetApp owner and administrator) to access visualized images to be displayed as part of Demo 2.

The three Virtual Network Functions (VNFs) composing the NetApp operate as follows:

**VNF: vPDC** (URLLC) manages the vPDC service that collects comparable measurement data from the PMUs located in the wide area of Greece and Bulgaria. The metrics pertain to an existent 400 kV interconnector. The two PMUs are installed in the substations connecting the opposite ends of the interconnector. In addition to collecting data under the C37.118 protocol [ IEEE C37.118 protocol] for comparison purposes, vPDC is specifically tasked to synchronize and validate measurements coming from different PMUs on both sides of the BG-GR (Bulgarian-Greek) border to deliver a real-time synchronized data stream. A considerable decrease of latency is achieved by virtualization of the PDC, which, *per se*, is brings it “closer” to the PMUs and limits integration costs.

**VNF: WAM Service** (URLLC) builds on the vPDC to provide several PMU status indicators and visualization functions. These functions may include, *inter alia*: a map showing current location of the PMU; firmware name, address, model, serial number, and version; rated system frequency (Hz) and actual measured value (frames per second); current and voltage phasor graph with hard real time updates; and voltage magnitude and angular difference monitor based on historical data for both monitored objects.

**VNF: Advisory Enabler** (URLLC) delivers RT consultancy system operation correction measures to the TSOs as well as for ex-post analysis in case of fault and contingency events. This service is based on a list of signals with alarm thresholds such as normal, critical or contingency. Once a threshold is reached, warnings or alarms are triggered depending on the abnormality detected. These can serve the SEE RSC to make informed advisory decisions on response measures or operational corrections that grid operators may consider for operational optimisation purposes.

**Figure 8.**
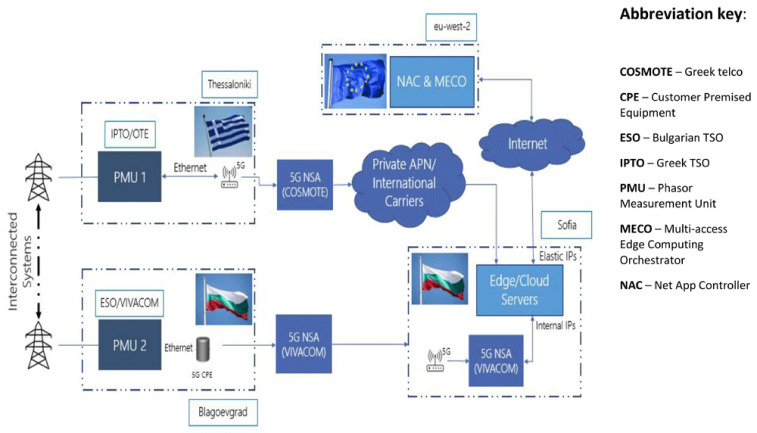
5G data flow of Demo 2 (Source: VIVACOM).

The high-level diagram for Demo 2 shown in Figure 8 illustrates the hierarchy interrelations between the energy infrastructure at Level 1 (PMUs installed in the wide area of Greece and Bulgaria), the edge cloud- based NetApp (MEC servers in telco data center), and the control rooms of both two TSOs at Level 2 where the three services are provided.

**Main actors and their roles in Demo 2**: RSC is the primary owning the WAM and servicing both TSOs (ESO and IPTO): TSO owns the transmission grid assets and responsible for ensuring grid stability and Security of Supply (SoS); Telecommunication Provider Telecommunication Provider owns, operates and maintains the Non |Standalone 5G network and its main responsibility is to ensure stable and reliable 5G coverage meeting the Service-Level Agreement (SLA) 5G network specifications; Infrastructure Owner providing the server that will host the OEP as well as the NetApps running at the edge; PMU measuring grid parameters ( *e.g.*, current and voltage magnitude and phase angle) with high-precision time synchronization.

**Demo scenarios** for the energy vertical service delivered by the **Demo 2 NetApp**:

- No system operation abnormalities found, no advisory suggestion offered.
- RoCoF deviation found, advisory suggestion sent to both TSOs.
- Voltage/current phasor abnormalities found, advisory suggestion sent to the affected TSO.

**5G network requirements for Demo 2:** URLLC service availability and reliability of 99.999%, URLLC E2E latency of 20-200 ms; vPDC absolute waiting time of 40 ms; Bandwidth 699-1500 kbps/node ^21^ Device Density of 1 Dev/km; High Security

**Key Performance Indicators (KPIs) for Demo 2**: E2E Latency (max 200 ms between user service endpoints), Reliability, Bandwidth, Network Slicing (URLLC), and vPDC Absolute Waiting Time.

**Figure 9.**
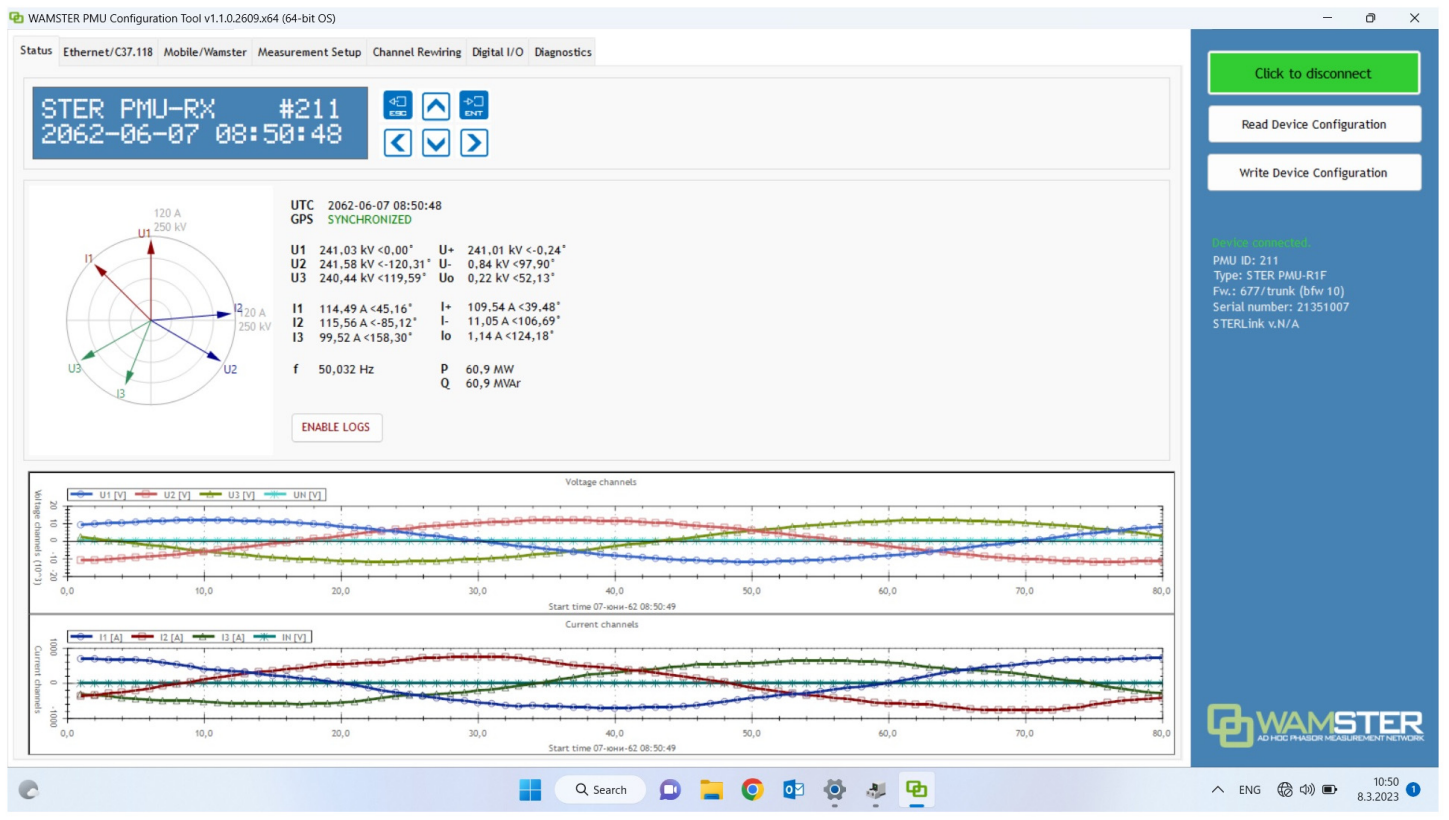
Graphical User Interface (GUI) of grid operator’s PMU with graphs and phasor diagrams.

The 5G data flow diagram for the 5G-enabled WAM service supported by Demo 2 is illustrated in Figure 8. The left side shows the telco infrastructure starting from the PMUs installed in both Blagoevgrad (BG) and Thessaloniki (GR) substations operated, correspondingly, by ESO and IPTO. The PMU in Blagoevgrad substation transmits measurement packs via 5G CPE (Customer Premises Equipment) through the 5G Non-standalone Network of the Bulgarian telco operator ^22^ to the edge cloud-based vPDC for further processing. At the same time, the PMU in Thessaloniki transfers its measurement packs using the 5G Non Standalone Network of the Greek telco operator. Incoming data from both PMUs are read, synchronized and validated by the edge cloud based vPDC with a waiting time of 40 ms, which then forwards it to the Advisory service whose main function is to provide early warning and suggestions to the TSOs for system security optimisations to improve prevention of critical grid conditions, such as power swings or frequency deviations, that could affect the interconnected power systems. The WAM service supports various PMU status indicators and visualization functions, including a map showing current location of the PMUs; firmware name, address, model, serial number, and version; rated system frequency and actual measured value (frames per second); current and voltage phasor graph with hard real time updates; voltage magnitude and angular difference based on historical data for both monitored substations. Figure 9 shows the Graphical User Interface of a PMU used in Demo 2.

**Figure 10.**
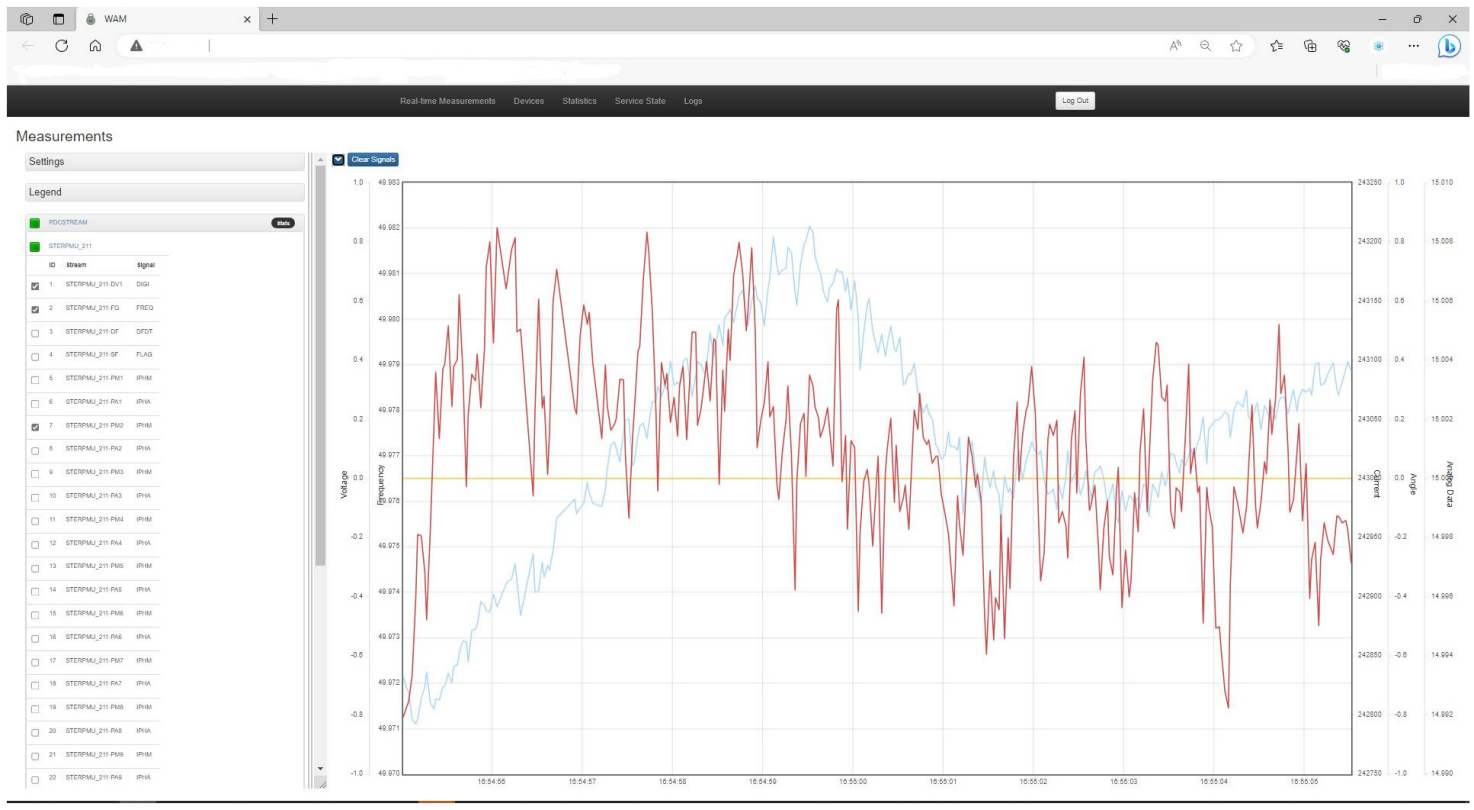
Wide Area Monitoring (WAM) service display of RT voltage, current and angle measurements.

The WAM NetApp display ( Figure 10) illustrates run-time measurements of critical parameters that the grid operator uses as part of Demo 2. The instance shown includes four RT measurement channels: voltage, current, phase angle, and frequency. Derivative products such as P (MW) and Q (MVar) and $\frac{df}{dt}$ may also be displayed.

The PMU supports sampling rate of 50 to 60 times per second and includes positive, negative, and zero sequences of voltage (U) and current (I) plus frequency (f), active (P) and reactive (Q) power. It collects this data from the energy infrastructure using measurement inputs ( *e.g.*, from current and voltage transformers and other sensors). Next, all RT PMU measurement data arrives at the WAM and vPDC Services in Vivacom Cloud. Then, upon validation and synchronization, it goes to the RSC-run Advisory Service NetApp for final processing and advisory to the grid operator depending on the type of abnormality found, if any. A non-exhaustive list of advisory services may include, *inter alia*, suggestions to suppress RoCoF deviation, voltage/current phasor abnormalities or power swings, recommendations for activation of Frequency Containment Reserves (FCRs), *etc.*

## Results

### Demo 1 Performance

**Figure 11.**
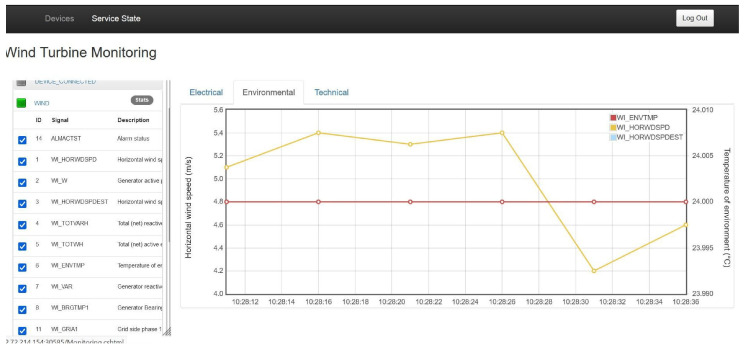
Demo 1 NetApp display.

Displays of the Demo 1 NetApp (VNF1 and VNF2) supporting real-time measurements of environmental, electrical and technical variables of the tested wind turbine are shown in Figure 11.

The actually measured E2E latency performance of Demo 1 reads as follows: Packets Sent = 50, Received = 50, Lost = 0 (0% loss), Approximate round trip times in milliseconds: Minimum = 8ms, Maximum = 47ms, Average = 18ms. This result was confirmed by series of ping and network ZABBIX monitoring tests performed at the edge cloud (MEC) server hosting the NetApp based in the Vivacom lab. It compares to that normally expected in a FO transmission network, falling clearly within the KPI range of 20–200 ms, while offering additional flexibility in terms of network slicing in the form of URLLC. No 5G network downtime was reported during the latency tests, confirming the set availability and reliability KPIs.

### Demo 2 Performance

The WAM&A NetApp runs on a cluster of edge cloud servers in Vivacom labs managed by Kubernetes, where the Docker images of the services are stored in a Docker Hub repository. From that repository, the WAM&A service is accessible to the RT-HIL testbed via web browser on a dedicated port with predefined credentials. In the RT HIL testbed, the vPDC receives phasor data from both PMUs and produces a data stream with synchronized and stamped measurements toward the WAM&A. For visualization of real-time interconnector performance, WAM receives a time-aligned PMU data input from the vPDC and generates graphs for the TSOs, which obtain current phasor that flows through the interconnector and the voltage phasors from the opposite ends. In the case of Advisory service, time aligned measurements received from vPDC are stored in a database and for statistical analysis purposes.

**Figure 12.**
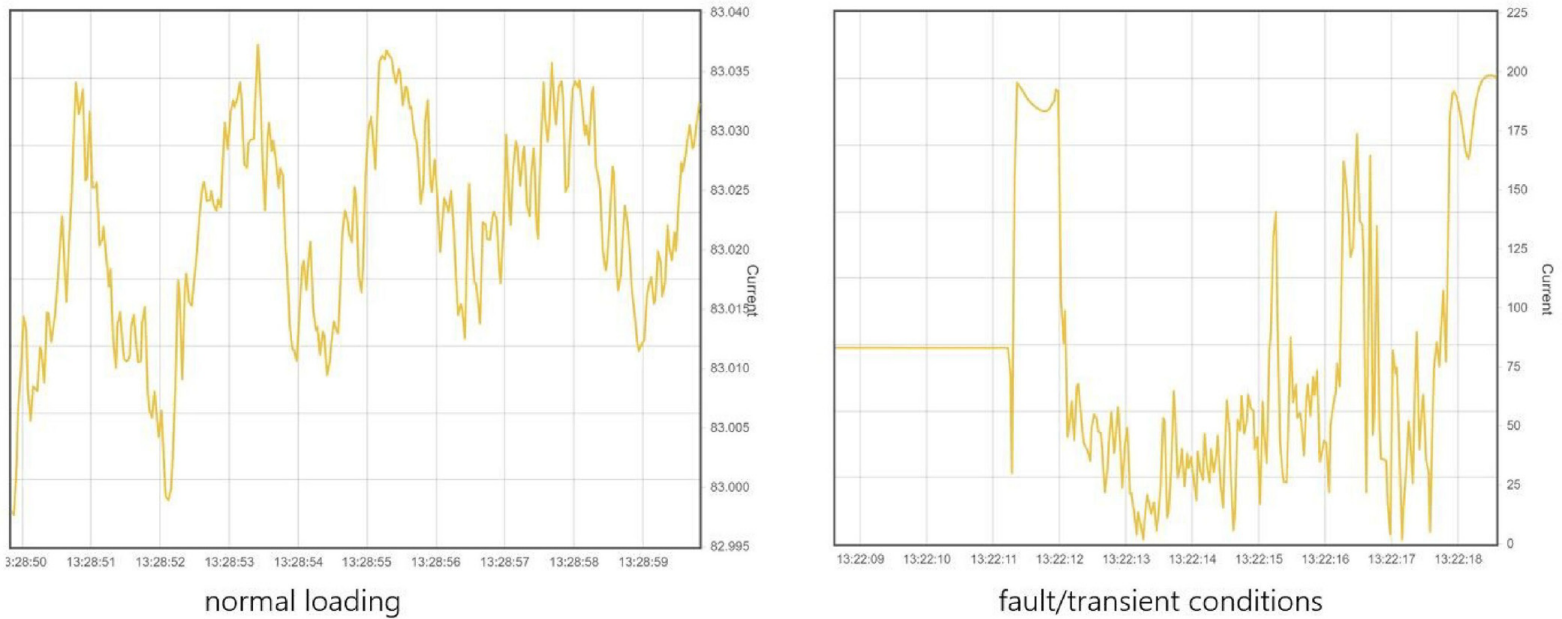
WAM Service Responsiveness.

This service monitors the tie line operating situation, which means that two relative case studies were run to verify that the NetApp adequately captures and monitors all operational conditions: (a) normal loading and (b) transients (fault event), as shown in Figure 12.

**Figure 13.**
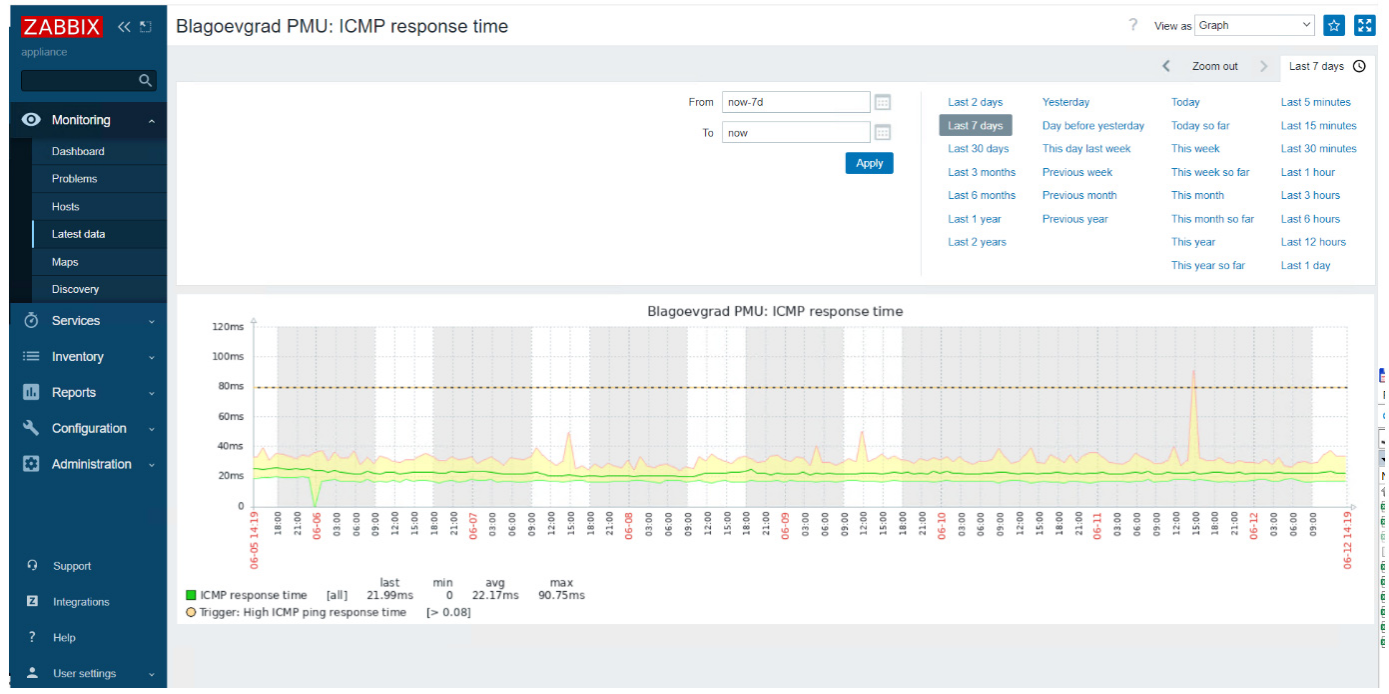
WAM Response Time Statistics for a 7-Day Continuous Recording.

The results of Demo 2 response time continuous recording of real-time PMU data stream in a 7-day period measured by Vivacom via ZABBIX network monitoring tool are reported in Figure 13.

According to the response time statistics, the average latency during the testing period was around 22 ms and the maximum value did not exceed 91 ms. It is demonstrated that, throughout the measurement recording time, only three spikes above 40 ms have occurred, two of them around 50 ms and one about 90 ms, which is way below the max 200 ms KPI taken as an established limitation criterion for transmission gird network delays. This reliability has previously also been confirmed by ZABBIX measurement statistics for a period of 30 days during which no interruptions, communication downtime or packet loss were identified, thus fulfilling the 99.999% availability and reliability performance requirements. 

**Figure 14.**
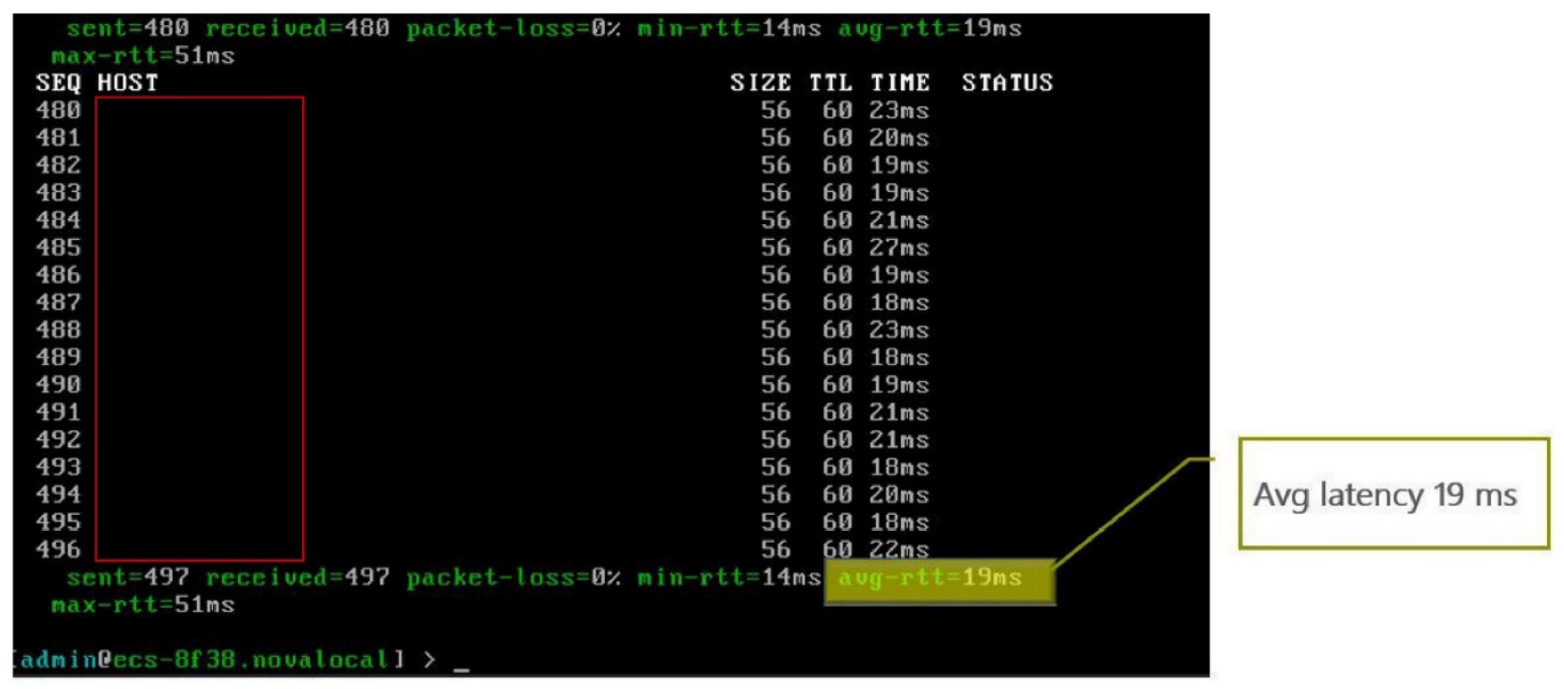
WAM E2E Latency Ping Results.

The results of series of E2E Latency ping tests at Vivacom MEC server hosting the WAM NetApp read as follows: minim latency 14 ms, max 51 ms, average 19 ms, and packet loss 0%, as evidenced in Figure 14.

The repeatedly measured E2E latency performance for Demo 2 was between 19 and 35 ms.

## Discussion

The concept and results reported in this work could support future developments of modularity and scale-up of WAM & A services across the energy vertical using by-design smart grid architectures.

In practical terms, as intrinsically volatile renewables tend to become dominant DRERs in a targeted complete decarbonization environment, power systems face unprecedented security and balancing challenges that need to be adequately addressed by accelerated and cost-effective integration of 5G communication technologies providing grid operators with ultrafast, low-latency and intelligent tools for higher visibility and control maneuverability of rapidly expanding distributed renewable energy resources. In addition to system security benefits at transmission and distribution level, smart grid solutions — as Demo 1— could also enable renewable generators to better forecast their maintenance needs and decrease both scheduled and accidental outages and downtime of their assets, leading to operational cost optimisations. This, in turn, also increases renewable generators’ scheduling precision and limit RES curtailment. It thus allows RES (potentially including DRER) to participate in the energy and balancing markets in a more productive manner, offering ancillary services to TSOs; avoiding penalties due to deviations from production schedules; and smoothing out unintentional and excessive power peaks usually seen at change of the hour, especially in intraday market timeframes.

In essence, 5G technology tailored for the energy vertical should not displace traditional FO and PLC (Power Line Communications) assets but rather offer, in a scalable and cost-effective way, critical flexibility as well latency optimisation and massive Machine-Type Communication (mMTC) for grid operators to be able to better monitor locations where such 5G energy vertical capabilities are most needed ( *i.e.*, in remote rural areas of massive RES expansion that FO and PLC communications fail to keep pace with and/or is too costly to build).

In system-wide terms, 5G-supported WAM capabilities as those demonstrated in Demo 2 represent a powerful tool for TSOs to boost their control area monitoring capacity and yield important added value on top of wire-bound communications commonly used in conventional power grids. Those benefits, as touched upon in the Demo 2 section, originate from the Smart5Grid tools (NetApps) that grid operators can leverage to monitor and control their synchronously interconnected control areas in a more efficient and preemptive manner, based on an URLLC and high granularity 5G architecture. Such tools allow grid operators to detect and suppress any abnormalities such as frequency deviations or power swings early enough (before their aggravation or escalation into contingencies).

## Conclusion

This work demonstrates the Smart5Grid Open Experimentation Platform’s latency minimisation capabilities by showcasing two pilot demos running on that platform whose measured performance in terms of real-time E2E latency, availability and reliability proves the viability of the proposed 5G-enabled energy vertical network architecture.

**For Demo 1:** The repeatedly measured average E2E latency of 18 ms compares to that of a fiber optic or wire-bound data network typically used in power grids, while offering additional flexibility in terms of network slicing in the form of latency minimisation, and falls within the KPI range of 20–200 ms as adopted at the 21st meeting of Working Group 5A of the International Telecommunication Union (ITU). This standard serves as a theoretical benchmark based on which the test results are interpreted. Demo 1 supports Run-Time Energy Production Monitoring and Predictive Maintenance Enabler services whose capabilities are demonstrated in the following video file: (download here). 

The NetApps developed and demonstrated under Demo 1 support a scalable solution for real-time (in ms latency) monitoring and improved maintenance of DRER. The 5G technology adds value by leveraging on advanced features like high device density support and cloud computing. Whilst Demo 1 pilot focuses on a wind farm, the demonstration activities with real data coming from a hydro power plant are also applicable by the NetApp for a variety of energy sources, including hydro and solar. 

**For Demo 2**: The repeatedly measured average E2E latency between the two field PMUs and the vPDC that runs on a Vivacom MEC server ranges between 19 ms and 35 ms, falling with sufficient reliability margin below the KPI of max 200 ms. This was confirmed by multiple ping tests at the Vivacom MEC server. The vPDC waiting time is 40 ms. The proposed WAM&A NetApp services conform to the following standard KPIs as adopted at the 21st meeting of Working Group 5A of the International Telecommunication Union (ITU): E2E Latency of 20–200 ms, vPDC absolute wait time within 40 ms, and bandwidth 699–1500 kbps/PMU. This standard serves as a theoretical benchmark based on which the test results are interpreted in this work. Demo 2 supports virtual Phasor Data Concentrator, Wide Area Monitoring, and Advisory (Enabler) services whose capabilities are demonstrated in the following video file: (download here). 

The NetApp developed and demonstrated under Demo 2 uses real-time measurements from two PMUs installed in the opposite ends of an existent 400 kV interconnector between Bulgaria and Greece. Those measurements were integrated in a RT HIL testbed and used to simulate normal and fault grid conditions, providing evidence of the underlying 5G-enabled OEP performance discussed in this work.

**For OEP:** Since both plot demos run on the Smart5Grid OEP, the above results demonstrate the platform’s claimed 5G-enabed latency PERFORMANCE, while the overall OEP architecture delivers flexibility, scalability, and modularity by design in addition to high availability and reliability to enhance observability and resilience of DRER-dominated power systems. 

## Disclaimer

The above key observations and conclusions expressed in this article are based on current physical data and results of the Smart5Grid project. Furthermore, as Smart5Grid is a still ongoing Horizon 2020 R&D project, the observations and conclusions contained in this article are neither definitive nor exhaustive. They may be subject to updates and revisions depending on the final qualitative and quantitative results of this project vs. KPIs.

## Ethics and consent

Ethical approval and consent were not required as part of this study.

## Data Availability

Dryad: Smart5Grid solutions for enhanced TSO grid observability and manageability in massive RES penetration environment. https://doi.org/10.5061/dryad.pvmcvdnq4 ( Shangov *et al.,* 2022) This project contains the following underlying data: Demo 1_NetApp_Timeseries_of_Realtime_PV_RES_Monitor_Results_1.xlsx Demo 2_NetApp_vPDC_Timeseries_Results.xlsx Dryad: Smart5Grid solutions for enhanced TSO grid observability and manageability in massive RES penetration environment. https://doi.org/10.5061/dryad.pvmcvdnq4 ( Shangov *et al.,* 2022) This project contains the following extended data: README_Smart5Grid_Article_Sustainable_Places_2022.txt Data are available under the terms of the CC0 1.0 Universal (CC0 1.0) Public Domain Dedication license. Zenodo: Smart5Grid solutions for enhanced TSO grid observability and manageability in massive RES penetration environment. https://doi.org/10.5281/zenodo.8058625 ( Shangov, 2022) This project contains the following extended data: Figure_1.jpg Figure_2.jpg Figure_3a.JPG Figure_3b.JPG Figure_4.jpg Figure_5.jpg Figure_6a.jpg Figure_6b.jpg Figure_7.jpg Figure_8.JPG Figure_9(1).jpg Figure_9(2).jpg Figure_9(3).jpg Figure_10.jpg Figure_11.jpg Figure_12.jpg Figure_13.jpg Figure_14.jpg SP2022_Manuscript_15090_Smart5Grid__Rev_4_Clean.docx Data are available under the terms of the Creative Commons Attribution 4.0 International license (CC-BY 4.0).

## Footnotes

^1^ https://github.com/argoproj/argo-workflows

^2^ https://www.keycloak.org

^3^ https://gitlab.com/gitlab-org/gitlab

^4^ https://goharbor.io

^5^ https://github.com/elastic/elasticsearch

^6^ https://github.com/elastic/logstash

^7^ https://github.com/elastic/beats

^8^ https://www.gridprotectionalliance.org

^9^ https://github.com/GridProtectionAlliance/gsf

^10^ https://github.com/GridProtectionAlliance/openPDC

^11^ https://github.com/GridProtectionAlliance/openHistorian

^12^ A phasor is a complex number representing a sine function whose amplitude (A), angular frequency (ω), and initial phase (θ) are time invariant. A common situation in electrical grids is the existence of multiple sinusoids, all having the same frequency but different amplitudes and phases. The only difference in their analytical representations is the complex amplitude (phasor). A linear combination of such functions can be included in the product of a linear combination of phasors (known as phase arithmetic) and the time/frequency dependent factor that common to all. Hence, a vPDC is a MEC/cloud based device that collects and synchronizes measurements from PMUs before transferring them

^13^ A Phasor Measurement Unit (PMU) is a devise that measures synchronized magnitude and phase angle values of an electrical phasor variable, *e.g.* current or voltage, in a power grid. Such synchronized measurements are known as synchrophasors. A PMU uses a common source of time synchronization such as Global Navigation Satellite System (GNSS) module/receiver and has wire- bound as well as wireless communication capabilities for data exchange with Supervisory Control And Data Acquisition (SCADA) systems.

^14^ HIL is a testing setup where real signals from a controller are connected to a testbed that mirrors (simulates) a real/physical system that the controller is tricked to consider as such instead of as a digital twin system. This enables running multiple test and experimentation scenarios as though a real system is being used but without the risks, costs and time associated with physical tests.

^15^ https://www.entra.energy/

^16^ In Demo 1, the wind farm consists of a single wind turbine with installed capacity of 2MW

^17^ The 5G communication requirements for both Demo 1 and Demo 2 are based on the 3GPP TS22.261 and ITU-T G.827 standards

^18^ www.eso.bg

^19^ www.admie.gr/en

^20^ Model and performance of the PMUs used for the Demo 2 demo: STERPMU-Rx, free standing unit, 32GB local flash memory, embedded STERLINK communication and GNSS module, 3Xu +3XI measurement terminals, GNSS receiver for time synchronization, 1xEthernet 10/100Mbps port, 1x4G modem, configuration tool

^21^ In the context of the bandwidth requirement, Nod refers a PMU connection point with the network.

^22^ https://www.vivacom.bg

## Software availability

The open Smart5Grid Open Experimentation Platform is developed using open-source software available from the following public links: https://github.com/argoproj/argo-workflows; https://www.keycloak.org; https://gitlab.com/gitlab-org/gitlab; https://goharbor.io; https://github.com/elastic/elasticsearch; https://github.com/elastic/logstash; https://github.com/elastic/beats

## References

[ref-2] Asprou M Akrytov A Hadjidemetriou L : The Impact of Wireless Communication Networks on Wide Area Monitoring and Protection Applications. *2022 IEEE International Smart Cities Conference.* Pafos, Cyprus, Sept,2022. 10.1109/ISC255366.2022.9922326

[ref-8] Deterministic frequency deviations – root causes and proposals for potential solutions, A joint EURELECTRIC – ENTSO-E response paper. Reference Source

[ref-3] IEEE standard for synchrophasors for power systems. 2011.

[ref-10] IEEE C37.118 protocol. Reference Source

[ref-6] Khan UN Yan L : Power Swing Phenomena and its Detection and Prevention.

[ref-1] Pau M Mirz M Dinkelbach J : A Service Oriented Architecture for the Digitalization and Automation of Distribution Grids.In: *IEEE Access.* 2022;10:37050–37063. 10.1109/ACCESS.2022.3164393

[ref-4] Sood VK Fischer D Eklund JM : Developing a communication infrastructure for the Smart Grid.In: *2009 IEEE Electrical Power & Energy Conference.*Montreal, Canada,2009. 10.1109/EPEC.2009.5420809

[ref-5] 5G PPP: The 5G Infrastructure Public Private Partnership. 2014. Reference Source

[ref-9] 5G for the Support of Smart Power Grids: Millisecond Level Precise Distributed Generation Monitoring and Real-Time Wide Area Monitoring.Part of the IFIP Advances in Information and Communication Technology book series (IFIPAICT).652. 10.1007/978-3-031-08341-9_1

[ref-11] Shangov D : Smart5Grid solutions for enhanced TSO grid observability and manageability in massive RES penetration environment. Dryad, [Data], 2022. 10.5061/dryad.pvmcvdnq4 PMC10521069 37767203

[ref-12] Shangov D : Smart5Grid solutions for enhanced TSO grid observability and manageability in massive RES penetration environment. *Zenodo* . 2022. 10.5281/zenodo.8058625 PMC10521069 37767203

[ref-7] TSI GS NFV: Network Function Virtualization (NFV); Terminology for main concepts in NFV. * Tech Rep ETSI GS NFV 003.*European Telecommunication Standards Institute (ETSI) (08 2018), v1.4.1. Reference Source

