# Supplementary material for: Smart5Grid Solutions for enhanced TSO grid observability and manageability in massive RES penetration environment
Source: Open Res Eur. 2023 Jul 26;3:18. Originally published 2023 Jan 27. [Version 2] doi: 10.12688/openreseurope.15090.2 (PMC10521069; doi:10.12688/openreseurope.15090.2)
Supplement: Peer review history

# Reviewer report: Reviewer response for version 2

**DOI:** 10.21956/openreseurope.17526.r34817

**Published:** 2023-09-18

**Authors:** Athanasios Bachoumis

**Affiliations:** 1 University of Patras, Patras, Greece

The paper presents experimental results for the SMART-GRID platform. In general, after the previous review rounds, the paper looks more comprehensive, with a strong narrative offering some tangible outcomes, especially in terms of E2E latency for two enabled energy use cases.

 I have the following comments to strengthen the paper further:

1. Please provide a reference for ZABBIX, the first time it is mentioned.
2. You refer through Inter-TSO RCS. Maybe you can rewrite it as such: through Regional Security Coordinators (RSC) in an area governed by two neighbouring TSOs.
3. Instead of WAMPC, as far as I know in the literature, the most used acronym is WAMPAC.
4. You state in the results of the first demo, that the mean E2E value is 18 ms. However, you state that the latency is in the range of 20–200 ms. Is that expected for fibre optic or the requirements you had for the demo? In the case of the second, please rewrite it, because in most cases it is lower than that range.
5. I have a question regarding the results for the second use case. Do you report the results of the real piloting data from the PMUs or through the HIL generated data? You need to make it clear from the beginning, because you also mention the real setup and it is a bit confusing.

Is the study design appropriate and does the work have academic merit?

Yes

Is the work clearly and accurately presented and does it cite the current literature?

Yes

If applicable, is the statistical analysis and its interpretation appropriate?

Yes

Are all the source data underlying the results available to ensure full reproducibility?

Yes

Are the conclusions drawn adequately supported by the results?

Yes

Are sufficient details of methods and analysis provided to allow replication by others?

Yes

Reviewer Expertise:

Smart grid applications

I confirm that I have read this submission and believe that I have an appropriate level of expertise to confirm that it is of an acceptable scientific standard.

# Reviewer report: Reviewer response for version 1

**DOI:** 10.21956/openreseurope.16318.r31780

**Published:** 2023-06-09

**Authors:** Stephen Okwudili Ugwuanyi

**Affiliations:** 1 University of Strathclyde, Glasgow, Scotland, UK

Horizon 2020 Smart5Grid Research and Innovation (R&I) *project findings *on the Open Experimentation Platform (OEP) for smart grid are presented in this research article. The article focuses on two use cases, the Millisecond level precise distribution generation control and Real-time wide area monitoring use cases for Transmission System Operators. An interesting project based on the research article, but its contents are mostly high-level details of the measurement platform components and implementation. The paper contains very useful information on the OEP platform design and implementation. However, it is presented in a technical report format and could be improved.

 The paper acts as a very good introduction to the Smart5Grid Project and to the use cases discussed. The title of the paper is “Smart5Grid Solutions for enhanced TSO grid observability and manageability in massive RES penetration environment”, yet the contribution on the observability and manageability is not clearly identified. There are limited results of the platform performance being included. Aside from the use cases, it is very unclear what the actual contribution of the article is. The authors can outline the paper's key contributions to prove the novelty or its significance within the introductory section. The paper needs to be restructured to align with the knowledge gap to be outlined.

 The research article is hard to follow because too many concepts are discussed. While key components of the platform like NetApps are presented, below are my comments/recommendations:

- The research article is more like a description of the overall project design and implementation, emphasising key components. As a result, the key contributions of the paper were not highlighted.
- The test results of 5G capabilities were not evidenced in the article, but 5G was mentioned severally in the paper to optimise, enhance and upgrade the legacy fibre optic and powerline communication in the OEP. Limited results have been shared to back up the claim.
- Regarding legacy fiber optic, more information is needed to evidence why it is considered legacy in the article.
- The local and public IP addresses shown in Figures 9 and 10 are potential cybersecurity breaches and should be removed or blurred.
- I could not easily identify the results of 5G slice impact on the OPE platform.
- On the “Methods”, the spelling of virtualisation is wrong (Vurtualisation).

To improve this paper, the authors could consider focusing the discussion more on the performance of the two use cases. While the project presented in the paper has merit, the information and presentation style differs slightly from standard research articles. Specifically, the methods and results sections need to be improved. Fewer details are provided on the results, like the type of 5G network and slice configurations.

 Also, the authors can substantially improve this paper by focusing the paper on specific aspects of the Smart5Grid OEP rather than its components, like analysing the performance (latency) of the 5G slice for the use cases. Latency minimisation is mentioned as one of the key objectives of Smart5Grid.

Is the study design appropriate and does the work have academic merit?

Partly

Is the work clearly and accurately presented and does it cite the current literature?

Partly

If applicable, is the statistical analysis and its interpretation appropriate?

Partly

Are all the source data underlying the results available to ensure full reproducibility?

Partly

Are the conclusions drawn adequately supported by the results?

No

Are sufficient details of methods and analysis provided to allow replication by others?

Partly

Reviewer Expertise:

Wireless communication, Network Security and Smart Grid Virtualisation

I confirm that I have read this submission and believe that I have an appropriate level of expertise to confirm that it is of an acceptable scientific standard, however I have significant reservations, as outlined above.

# Response

**Published:** 2023-06-20

**Authors:** Daniel Shangov

Dear Stephen Ugwuanyi,  Thank your for taking your time to review version 1 of this paper. Having reflected on your constructive comments and recommendations, I have now posted version 2 trying to reflect them to the extent possible. Please find below my reponses bullet by bullet:

- The research article is more like a description of the overall project design and implementation, emphasising key components. As a result, the key contributions of the paper were not highlighted.

***Author's response**: The structure is now changed from a more deliverable-style report to a research article consisting of **Abstract** (concise summary of the research objective, methods, and key findings), **Introduction** (overall context, motivation for the study, and definition of the research objective – focusing on the Smart5Grid latency performance supported by Demo 1 and Demo 2), Related Works (references of related previous research and literature), **Methodology** (experimental setup, testbed, and data collection methods), **Results** (the outcome of series of network monitoring probes and ping tests are reported in textual and graphic form to support the OEP latency performence thesis); **Discussion** (interpretation of the results and discussion of potential future works based on the findings), and **Conclusion** (interpretation of the results versus the research objective, referenced literature, and theoretical basis.). Note that, for the purpose for this article and because it does not discuss UC1 and UC2, I have renamed UC3 and UC4 to Demo 1 and Demo 2 correspondingly. After this redesign, the UC-style is now replaced with a more research article-type of paper.  The contribution of this work is now mentioned in the sense of proposed 5G-enabled latency oprimisation solutions for the energy vertical and, in particulary, for power grid dominated by decentralised renewable energy deployment and its implications for interconnected power systems.*

- The test results of 5G capabilities were not evidenced in the article, but 5G was mentioned severally in the paper to optimise, enhance and upgrade the legacy fibre optic and powerline communication in the OEP. Limited results have been shared to back up the claim.

***Author's response**: The latency performance of the 5G-enabled Open Experimentation Platform (OEP) is now supported with the results of a number of network monitoring statistics and ping tests as detailed in the Methodology section. *

- Regarding legacy fiber optic, more information is needed to evidence why it is considered legacy in the article.

***Author's response**: The term 'legacy' was actually meant as 'existent' rather than 'obsolete'. Nevertheless, to avoid misunderstanding, this term is now changed for 'existent' or 'well-established'. It describes the fact that fiber opric is the most widespread means of communication in modern power grids. The paper explores how the solutions could complement and add value to existent FO assets instead of replacing them. *

- The local and public IP addresses shown in Figures 9 and 10 are potential cybersecurity breaches and should be removed or blurred.

***Author's response**: Both figures were revised accordingly and the IP addresses are now blurred. *

- I could not easily identify the results of 5G slice impact on the OPE platform.

***Author's reponse**: As 5G network slicing generally includes massive Machine Type Communication (mMTC), Ultra Reliable Low Latency Communication (URLLC), and enhanced Mobile Broadband (eMBB), this paper focuses on the latency minimisation offered by the OEP in terms of Ultra Reliable Low Latency Communication (URLLC) slice. This is now supported by test readings reported in the Results section.*

- On the “Methods”, the spelling of virtualisation is wrong (Vurtualisation).

***Author's response**: Proof-reading was done in an attempt to fix as many spelling issues as possible, including 'Vurtualisation' in the Methods section.*

 Thanks and kind regards, Daniel Shangov, Corresponding Author

# Reviewer report: Reviewer response for version 1

**DOI:** 10.21956/openreseurope.16318.r31783

**Published:** 2023-05-25

**Authors:** Younes Balboul

**Affiliations:** 1 Sidi Mohamed Ben Abdellah University, Fes, Morocco

This research article presents the findings and implications of the Horizon 2020 Smart5Grid Research and Innovation (R&I) project. The article focuses on the development of the Smart5Grid Open Experimentation Platform (OEP) and its application in two key Use Cases (UCs) for Transmission System Operators (TSOs). The research showcases the NetApps developed within the project and their contribution to improving the efficiency, reliability, and flexibility of legacy wire-bound communication in transmission and distribution grids.

 However, it is important to note that this research article is written as a technical specification report and does not adhere to the conventional structure and organization of a typical research article. To improve the overall structure and flow of the article, it is recommended that the authors follow the standard structure of a research article, which typically includes sections such as Abstract, Introduction, Related Works, Methodology, Results, Discussion, and Conclusion.

 In terms of Related Works, the article briefly discusses the background and context of the Smart5Grid project. However, a more comprehensive review of relevant literature and previous research is needed to situate the project within the broader research landscape and clearly identify the specific knowledge gap being addressed.

 Regarding the Results section, the article should provide clear measurements and compare the obtained values with standard or accepted average values for similar systems. This would enhance the credibility and applicability of the reported results.

 To improve the article, it is recommended to adhere to the conventional structure of a research article, which typically includes the following sections:

- Abstract: Provide a concise summary of the research objective, methodology, key findings, and implications.
- Introduction: Present the background, context, and motivation for the study, including a clear statement of the research problem or objective.
- Related Works: Review relevant literature and previous research that informs the current study, highlighting the knowledge gap addressed by the research.
- Methodology: Describe the research design, data collection methods, experimental setup, and analytical techniques employed in the study.
- Results: Present the findings obtained from the analysis of the collected data or the outcomes of the experimental study. Include clear measurements and compare them to established standards or average values.
- Discussion: Interpret and analyze the results, relating them to the research objective, existing literature, and theoretical framework. Address any limitations or challenges encountered during the study.
- Conclusion: Summarize the main findings, discuss their implications, and suggest future research directions or potential applications.

By organizing the article according to this standard structure and incorporating the suggested improvements, the authors can effectively communicate their research findings, enhance the credibility of the results, and facilitate readers' understanding of the study's contribution.

Is the study design appropriate and does the work have academic merit?

Partly

Is the work clearly and accurately presented and does it cite the current literature?

Partly

If applicable, is the statistical analysis and its interpretation appropriate?

No

Are all the source data underlying the results available to ensure full reproducibility?

Partly

Are the conclusions drawn adequately supported by the results?

No

Are sufficient details of methods and analysis provided to allow replication by others?

Partly

Reviewer Expertise:

5G and 6G NetworksCloud InfrastructureIoT Technologies

I confirm that I have read this submission and believe that I have an appropriate level of expertise to confirm that it is of an acceptable scientific standard, however I have significant reservations, as outlined above.

# Response

**Published:** 2023-06-20

**Authors:** Daniel Shangov

Dear Younes Balboul,  Thank you for having peer-reviewed version 1 of this paper. Your valuable recommendations are now reflected in version 2. Overall, the work was restructured from a deliverable/technical desription to a research paper, as explained below bullet by bullet: 

- Abstract: Provide a concise summary of the research objective, methodology, key findings, and implications.

***Author's respnse**: **Abstract** is now included (synopsis of research objective, discussed methods, findings, and potential impact).*

- **Introduction**: Present the background, context, and motivation for the study, including a clear statement of the research problem or objective.

***Author's respnse**: **Introduction** is now included (with background, motivation and definition of the research objective – focusing on the Smart5Grid latency performance supported by Demo 1 and Demo 2).*

- Related Works: Review relevant literature and previous research that informs the current study, highlighting the knowledge gap addressed by the research.

***Author's response: Related Works **are now included (with references of related previous research and literature of relevance to the subject of research).*

- Methodology: Describe the research design, data collection methods, experimental setup, and analytical techniques employed in the study.

***Author's response: Methodology** is now included (with visual and textial explainations of experimental setup, testbed, and data collection methods).*

- Results: Present the findings obtained from the analysis of the collected data or the outcomes of the experimental study. Include clear measurements and compare them to established standards or average values.

***Author's response: Results **are now reported (diagrams, network monitoring statistics, ping test results supporting the claimed latency performance).*

- Discussion: Interpret and analyze the results, relating them to the research objective, existing literature, and theoretical framework. Address any limitations or challenges encountered during the study.

***Author's response: Discussion **is incorporated (the results are interpreted from power grid perspective in massive RES rollout conditions, future developments/applications based on the findings are discussed as well).*

- Conclusion: Summarize the main findings, discuss their implications, and suggest future research directions or potential applications.

***Author's response: Conclusion **is now available too (with interpretation of the results versus research objective and reference standard such as KPIs). Note that, for the purpose for this article and because it does not discuss UC1 and UC2, I have now renamed UC3 and UC4 to Demo 1 and Demo 2, correspondingly. Any reference to other Use Cases have been completely eliminated.  After this redesign, the UC-style is now further replaced with a more research article-type of paper.*

 Kind regards and thank you for reviewing the second version of this work as well.  Daniel Shangov, Corresponding Author

# Reviewer report: Reviewer response for version 1

**DOI:** 10.21956/openreseurope.16318.r30738

**Published:** 2023-03-20

**Authors:** Carlos Jesús Bernardos Cano

**Affiliations:** 1 Universidad Carlos III de Madrid, Madrid, Spain

The paper reports on the results from some use cases addressed within the framework on a European project. The problem tackled is quite interesting and is related to a vertical for which 5G has shown to enable significant enhancements.

 I have some concerns/comments with the current form of the paper (listed below in no particular order). I think the authors should address those for the paper to be considered for indexing:

- The paper makes an extensive use of acronyms. Most of them are properly expanded the first time they are used, but the paper is still quite difficult to follow because of that.
- While I see and understand that this venue is very much for contributions from EU projects, I think in the current narrative of the paper, it is very much deliverable-style. I’d recommend not to follow UCX style and just describe the two use cases addressed in the paper.
- Figures need work to make them readable. Resolution is too low in most of the cases and it seems that they have been copied and pasted as an image from a Microsoft product (some showing the red underlining used to mark a spelling error).
- The paper lacks details on the actual architecture and software used. More details regarding the orchestration used and the 5G network are needed. As an example, it is not explained what a “signal emulator” is and how it is used. Another example, what is “5.208.x.x” in Figure 3?
- The paper indicates several times that latencies of 10ms are obtained, but no details are provided. I’d expect detailed tables and/or figures with obtained experimental results. Is legacy 5G used over a commercial network or an experimental network? URLLC is mentioned to be used, but this is not often available in commercial networks.
- Some typos are found in the paper that need to be fixed.

Is the study design appropriate and does the work have academic merit?

Partly

Is the work clearly and accurately presented and does it cite the current literature?

Partly

If applicable, is the statistical analysis and its interpretation appropriate?

No

Are all the source data underlying the results available to ensure full reproducibility?

Partly

Are the conclusions drawn adequately supported by the results?

Partly

Are sufficient details of methods and analysis provided to allow replication by others?

No

Reviewer Expertise:

5G, network virtualization, experimental validation

I confirm that I have read this submission and believe that I have an appropriate level of expertise to state that I do not consider it to be of an acceptable scientific standard, for reasons outlined above.

# Response

**Published:** 2023-06-20

**Authors:** Daniel Shangov

Dear Carlos Jesus Bernardos Cano,  Let me start by thanking you for your constructive peer-review comments, which I believe will help refining this work. Please note that I have now submitted a revised version 2 of the article aimed at reflecting them as much as possible and applicable. Below are my responses to your improvement suggestions: 

- The paper makes an extensive use of acronyms. Most of them are properly expanded the first time they are used, but the paper is still quite difficult to follow because of that.

***Author's response**: Revisions were made in version 2 to handle the use of acronyms in a way that facilitates the reading process. Nevertheless, due to the specificity of the platform and related pilot demos discussed in this paper, the number of acronyms is still relatively high. However, acronyms are now explained (written out) as frequently as needed to smooth out comprehension. *

- While I see and understand that this venue is very much for contributions from EU projects, I think in the current narrative of the paper, it is very much deliverable-style. I’d recommend not to follow UCX style and just describe the two use cases addressed in the paper.

***Author's response**: The paper in now restructured from a techical description or a deliverable-style into a research-focused article consisting of **Abstract **(synopsis of research objectives, methods, key reported findings); **Introduction **(context, motivation of the study, and definition of the research objective - now focusing on the 5G-enabled Open Experimentation Platform's (OEP) latency perfomance examined and tested in two Demos as detailed in the paper); **Related Works** (referring to previous research and literature of relevance to the subject), **Methodology **(descriptions of the experimental setup and data collection methods - including the open software used to develope the OEP as well as the overall architecture and testbed of the two Demos committed to substantiate the latency performance claims), **Results **(detailed report of the test results and netrowk monitoring statistics in both textual and graphic form);  ( **Discussion** (interpretation of the results and deliberation of potential future works/developments based on reported findings), and **Conclusion **(interpretation of the resuls versus research objective, reference standard, and theoretical basis). Moreover, the UC-style was eliminated by stressing on Demo 1 (previously called UC3) and Demo 2 (previously called UC4) as part of the experimental setup for this research, while UC1 and UC2 were eliminated whatsoever as irrelevant for this paper. *

- Figures need work to make them readable. Resolution is too low in most of the cases and it seems that they have been copied and pasted as an image from a Microsoft product (some showing the red underlining used to mark a spelling error).

***Author's response**: Well noted. Hence, some figures of substandard quality were removed and others were revised to improve their quality in line with ORE's formatting and resolution requirements. Now figures are hupefully more stremlined and reader-frendly. *

- The paper lacks details on the actual architecture and software used. More details regarding the orchestration used and the 5G network are needed. As an example, it is not explained what a “signal emulator” is and how it is used. Another example, what is “5.208.x.x” in Figure 3?

***Author's response**: The software that was used to develop both the OEP and the Demos of reference was described in more detai in the Methodology section along with additional elaboration (as additional figurs and wording) of the underlying 5G network and overall orchestration arrangement. For instance, ambiguouls references such as “5.208.x.x” in Figure 3 (now revised and replaced) are now dealt with. Also, it is explaned what a 'signal emulator' does, i.e. in Demos 1 and 2 it creates a digital twin of a real grid element to test its 5G-enabled network perormance.*

- The paper indicates several times that latencies of 10ms are obtained, but no details are provided. I’d expect detailed tables and/or figures with obtained experimental results. Is legacy 5G used over a commercial network or an experimental network? URLLC is mentioned to be used, but this is not often available in commercial networks.

***Author's response**: Pertaining to the “10ms“ latency reported, kindly note that this has been a one-time current measurement only and is therefore now completely removed from the paper as having been mistakenly included in the first place. Instead, **to offer a more solid experimental backup of the 5G-enabled lateny thesis, extensive measurement statistics and results are now summarised and reported in the Results section**, as detaied in the Demos' testbed and methodology descriptions. *

- Some typos are found in the paper that need to be fixed.

***Author's response**: Proof-reading was performed in an attemt to fix as many pending spelling issues as possiible.  Thank you in advance for reviewing the revised verion 2 as well.*

*Kind regards, Daniel Shangov, Corresponding Author*
